# Integrative physiological and transcriptome analyses provide insights into the Cadmium (Cd) tolerance of a Cd accumulator: *Erigeron canadensis*

**DOI:** 10.1186/s12864-022-09022-5

**Published:** 2022-11-28

**Authors:** Chenchen Gan, Zhaochao Liu, Biao Pang, Dan Zuo, Yunyan Hou, Lizhou Zhou, Jie Yu, Li Chen, Hongcheng Wang, Lei Gu, Xuye Du, Bin Zhu, Yin Yi

**Affiliations:** 1 School of Life Sciences, Guizhou Normal University, Guiyang, 550025 People’s Republic of China; 2 School of Advanced Agriculture and Bioengineering, Yangtze Normal University, Chongqing, 408100 People’s Republic of China

**Keywords:** *Erigeron Canadensis*, Transcriptome, Cadmium stress, Accumulator, Physiology

## Abstract

Cadmium (Cd) is a highly toxic pollutant in soil and water that severely hampers the growth and reproduction of plants. Phytoremediation has been presented as a cost-effective and eco-friendly method for addressing heavy metal pollution. However, phytoremediation is restricted by the limited number of accumulators and the unknown mechanisms underlying heavy metal tolerance. In this study, we demonstrated that *Erigeron canadensis* (Asteraceae), with its strong adaptability, is tolerant to intense Cd stress (2 mmol/L CdCl_2_ solution). Moreover, *E. canadensis* exhibited a strong ability to accumulate Cd^2+^ when treated with CdCl_2_ solution. The activity of some antioxidant enzymes, as well as the malondialdehyde (MDA) level, was significantly increased when *E. canadensis* was treated with different CdCl_2_ solutions (0.5, 1, 2 mmol/L CdCl_2_). We found high levels of superoxide dismutase (SOD) and ascorbate peroxidase (APX) activities under 1 mmol/L CdCl_2_ treatment. Comparative transcriptomic analysis identified 5,284 differentially expressed genes (DEGs) in the roots and 3,815 DEGs in the shoots after *E. canadensis* plants were exposed to 0.5 mM Cd. Functional annotation of key DEGs indicated that signal transduction, hormone response, and reactive oxygen species (ROS) metabolism responded significantly to Cd. In particular, the DEGs involved in auxin (IAA) and ethylene (ETH) signal transduction were overrepresented in shoots, indicating that these genes are mainly involved in regulating plant growth and thus likely responsible for the Cd tolerance. Overall, these results not only determined that *E. canadensis* can be used as a potential accumulator of Cd but also provided some clues regarding the mechanisms underlying heavy metal tolerance.

**Supplementary Information:**

The online version contains supplementary material available at 10.1186/s12864-022-09022-5.

## Introduction

Heavy metal pollution, such as that by cadmium (Cd), lead (Pb), copper (Cu), manganese (Mn), and nickel (Ni), has become a widespread and urgent problem due to the expansion of industrialization and urbanization [1]. Among these contaminants, Cd, which is usually present in water and soil in the form of ions (Cd^2+^), has become one of the most serious contaminants because of its wide range and high mobility in soil‒plant systems [2]. It is believed that ingestion of excessive Cd, which can accumulate via the food chain, poses severe threats to human health by causing various diseases, such as cancer and kidney and lung dysfunction [3]. Therefore, it is crucial to reduce or even remove Cd from contaminated soil and water [4].

To remove Cd from contaminated soil and water, physical and chemical remediation schemes have been carried out for several decades [5]. However, physical and chemical remediation is usually expensive and has harmful effects on soil and water properties in practice [6, 7] and may even introduce new pollutants into soil and water. Recently, phytoremediation using plants, which has been presented as a cost-effective and eco-friendly method for solving heavy metal pollutants, has become increasingly valued [8–10]. Generally, the phytoremediation of Cd-contaminated soil and water depends on accumulators that exhibit a strong ability to accumulate Cd and a strongly enhanced ability for Cd detoxification [11, 12]. However, these Cd accumulators are quite limited. The hyperaccumulator database (www.hyperaccumulators.org) includes global information on hyperaccumulators, and only a single-digit number of species were identified as hyperaccumulators of Cd. Moreover, some species defined as hyperaccumulators or accumulators show small biomasses and weak adaptability [13], which further restricts their application in the phytoremediation of contaminated soil and water.

It is widely known that Cd generally induces reactive oxygen species (ROS) accumulation, which severely inhibits growth and even leads to plant death [14–16]. The ROS level has been utilized as an index of the degree of harm in plants. Heavy metals usually cause damage to plants by directly or indirectly increasing ROS levels [17]. The increase in ROS levels under Cd stress hampers the photosynthetic capabilities of plants [18, 19]. Moreover, excessive production of ROS leads to sharply increased lipid peroxidation, which may cause cell death [20]. Proline (Pro) can reduce free radical levels and lipid peroxidation and improve membrane integrity, helping cells cope with NaCl-induced oxidative damage in *Eurya emarginata* plants [21]. Some antioxidant enzymes, such as superoxide dismutase (SOD), peroxidase (POD), glutathione reductase (GR), and ascorbate peroxidase (APX), are also used in defence strategies by plants to protect themselves from ROS [22].

In addition, several transporter genes have been shown to be involved in Cd accumulation in plants [23]. A dominant transporter, Nramp5 (natural resistance associated macrophage protein 5), has been recognized as an important transporter for root uptake in rice and barley. Cd concentrations were significantly reduced in the roots, shoots, and seeds of rice after knocking out *Nramp5* using RNAi or CRISPR/Cas9 [24–26]. Furthermore, Nramp1 has been demonstrated to be prominently involved in the uptake of Cd from soil [27–29]. After uptake by the roots, some heavy metal ATPases (HMAs), including HMA1, HMA2, and HMA3, are involved in Cd translocation and accumulation [30–32]. Moreover, some transporters, such as LCT1 (low-affinity cation transporter 1) and MTL (metallothionein-like protein), are essential elements that promote Cd transport and accumulation [33–35].

*Elodea canadensis* (Compositae), also known as *Conyza canadensis*, is an annual plant with a large biomass that is native throughout most of North and Central America. *E. canadensis* has invaded most areas in China due to its strong adaptability and rapid reproduction. Wei et al. [36] showed that *E. canadensis* can be used as an accumulator of Cd. Subsequently, several studies on the physiological responses to Cd stress reconfirmed the Cd tolerance of this plant and its potential value [37, 38]. However, the molecular mechanisms underlying heavy metal tolerance in *E. canadensis* have not been addressed. In this study, phenotypic and physiological analyses were conducted to investigate the Cd detoxification capability of an *E. canadensis* line collected from a mercury mining area. These results demonstrated that this *E. canadensis* line can be used as an accumulator of Cd. Transcriptome analysis was then employed to decipher the potential molecular mechanisms underlying this heavy metal tolerance. This study helps to elucidate the mechanism of Cd tolerance in *E. canadensis* and provides a reference for further exploration of Cd tolerance in Asteraceae species.

## Results

### The tolerance and accumulation of Cd in *E. canadensis* under different Cd concentrations

**Fig. 1:**
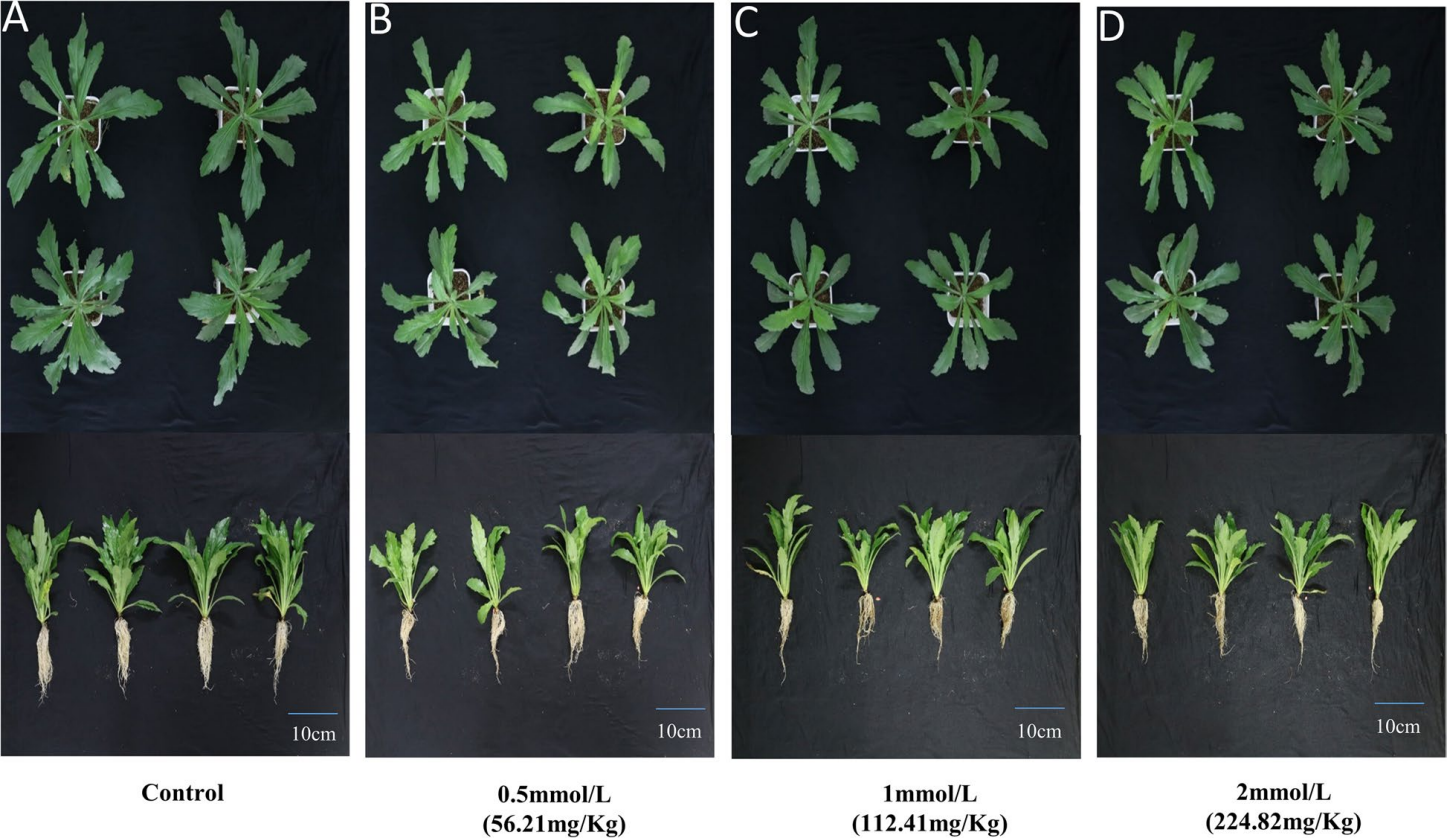
Phenotype of *E. canadensis* treated with different CdCl_2_ solutions. **A**: control plants; **B**-**D**: plants treated with 0.5 mmol/L, 1 mmol/L, and 2 mmol/L CdCl_2_ solutions

After being treated with different concentrations of CdCl_2_ for seven days, *E. canadensis* plants were harvested to determine their tolerance to Cd stress. Compared to the control plants, the plants treated with 0.5 mmol/L CdCl_2_ had fewer roots and lower biomass (Fig. 1). However, the plants treated with different concentrations of CdCl_2_ did not show significant differences in plant architecture (Fig. 1B-C). Moreover, no wilting or dead leaves were observed in the treated plants, indicating that the *E. canadensis* plants were tolerant to intense Cd stress.

**Fig. 2:**
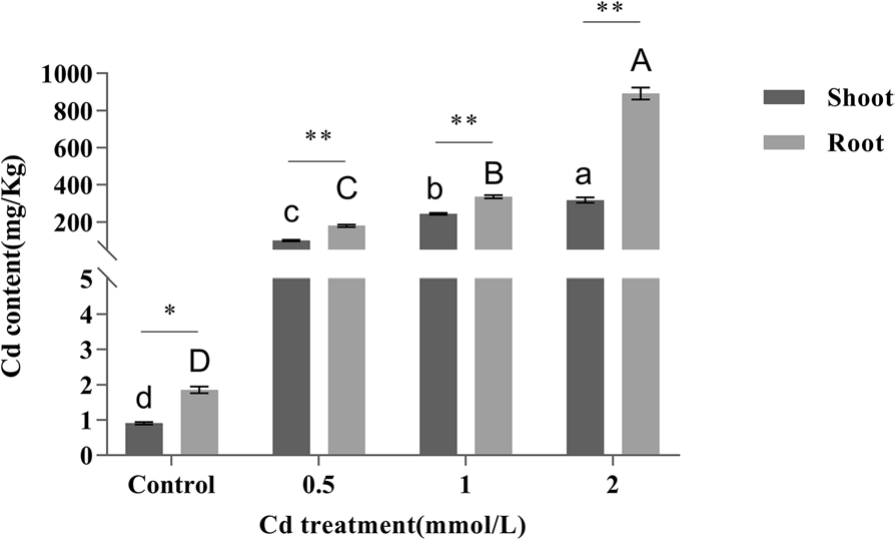
Cd content in roots and shoots of control and treated *E. canadensis* plants. The uppercase and lowercase letters indicate significantly different groups in roots and shoots, respectively (t test, *p* < 0.05). The symbols * and ** indicate that the two groups were significantly (t test, *p* < 0.05) and extremely significantly (t test, *p* < 0.01) different, respectively

Additionally, we measured the levels of Cd in both roots and shoots of the control and treated plants to determine the Cd accumulation ability of *E. canadensis*. The results (Fig. 2; Table S1) showed that the treated plants had a strong ability to accumulate Cd. The Cd content in all the roots and leaves of the treated *E. canadensis* plants approached or exceeded 100 mg/kg, which has been recognized as the cut-off for hyperaccumulators of Cd [39]. Briefly, the Cd content in the dry matter of roots and shoots was 178.72 ± 6.23 mg/kg and 99.11 ± 3.93 mg/kg, 335.55 ± 8.80 mg/kg and 243.83 ± 5.00 mg/kg, and 891.80 ± 32.86 mg/kg and 317.57 ± 14.85 mg/kg under treatment with 0.5 mmol/L, 1 mmol/L, and 2 mmol/L CdCl_2_ solutions, respectively. The Cd enrichment factor (shoot-to-medium concentration ratio) was 1.76, 2.17, and 1.41 under the 0.5 mmol/L, 1 mmol/L, and 2 mmol/L CdCl_2_ treatments, respectively. However, the Cd translocation factor (shoot-to-root concentration ratio) did not exceed 1.0 (the critical value for heavy metal hyperaccumulators) in the Cd treatments (0.55, 0.73, and 0.36 under 0.5 mmol/L, 1 mmol/L, and 2 mmol/L CdCl_2_, respectively).

### Physiological responses to Cd stress in *E. canadensis*

**Fig. 3:**
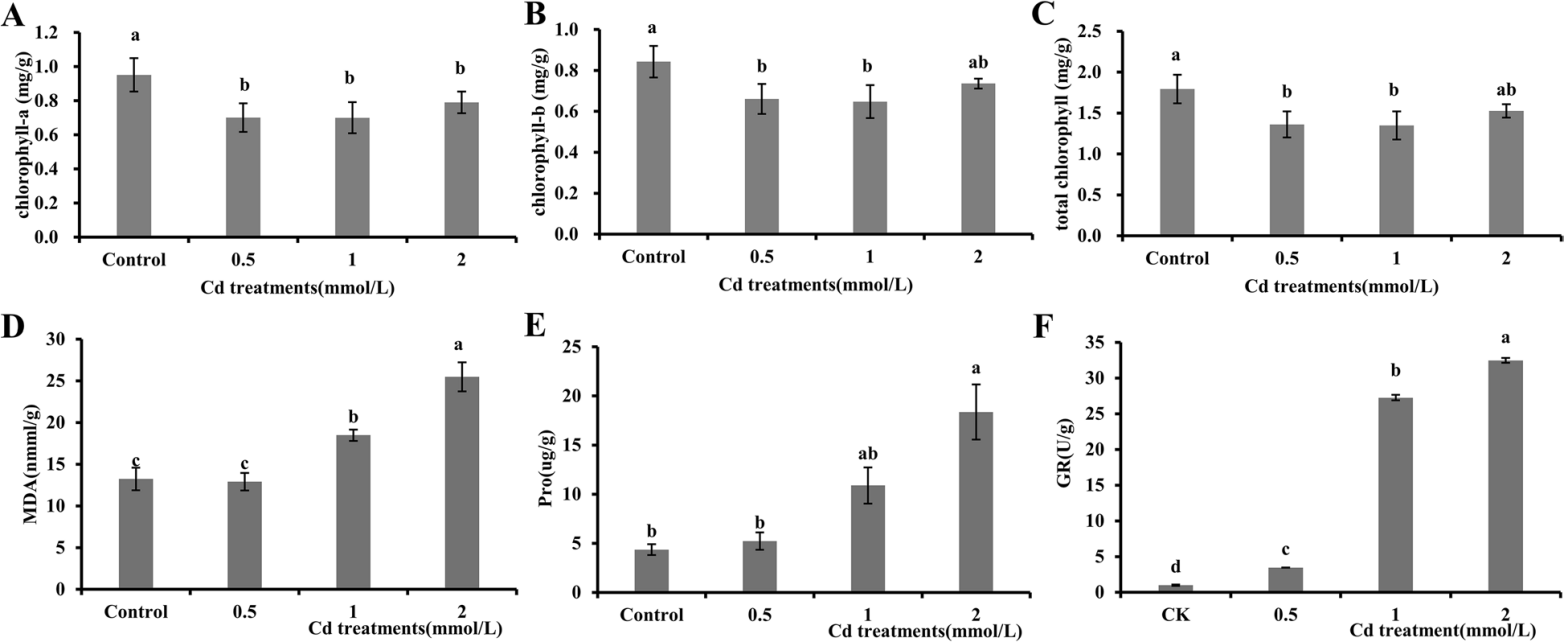
Chlorophyll, MDA, and Pro levels and GR activity in *E. canadensis* shoots under different CdCl_2_ treatments. Each value represents the mean ± SE of nine individual replicates. The data with the same letters are not significantly different at *p* < 0.05 (t test). The error bars indicate the standard error (SE) of the mean

Some physiological indices, including the chlorophyll content, Pro content, MDA content, GR activity, and antioxidant enzyme activities, were also measured. Compared to those of the control, the total chlorophyll, chlorophyll a, and chlorophyll b levels in leaves decreased under the different CdCl_2_ treatments (Fig. 3A-C). However, we found that the chlorophyll b content in the 2 mmol/L CdCl_2_ treatment was comparable to that in the control (t test, *p* > 0.05). The MDA content, which has been used as an indicator for lipid peroxidation in plants, was significantly increased after treatment with 1 mmol/L and 2 mmol/L CdCl_2_ but did not show obvious differences under 0.5 mmol/L CdCl_2_ treatment compared to that in the control (*p* > 0.05; Fig. 3D). Similar results were observed for the Pro content (Fig. 3E). Intriguingly, a more pronounced effect on GR activity was observed, wherein the GR activity was several times higher under Cd stress than in the control (Fig. 3F).

**Fig. 4:**
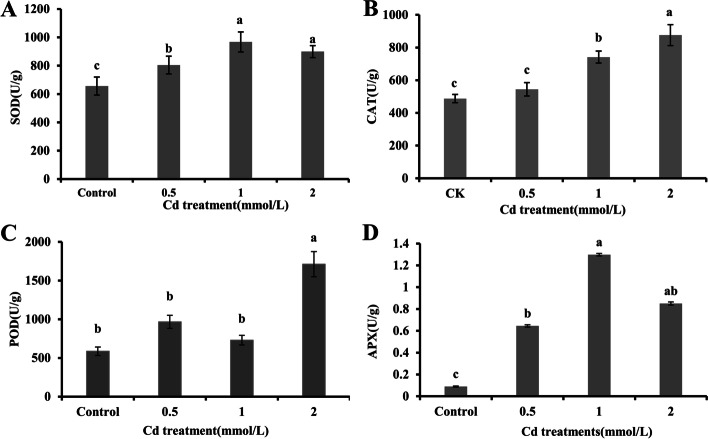
Antioxidant enzyme activities (SOD, POD, CAT, and APX**)** in *E. canadensis* shoots under different CdCl_2_ treatments. Each value represents the mean ± standard error (SE) of nine individual replicates. The data with the same letters are not significantly different at the cut-off of *p* < 0.05 (t test)

The SOD activity increased markedly in shoots treated with the different CdCl_2_ solutions (*p* < 0.05; Fig. 4A). However, the SOD activity in the shoots under the 2 mmol/L CdCl_2_ treatment was slightly lower than that under the 1 mmol/L CdCl_2_ treatment (*p* > 0.05). The CAT activity showed an increasing trend after Cd treatment (Fig. 4B). The greatest CAT activity was 179.6% in shoot tissues under the 2 mM Cd treatment. This result implied that *E. canadensis* has a strong ability to cope with Cd-induced oxidative stress. Intriguingly, the POD activity did not show an obvious increase under the 0.5 mmol/L and 1 mmol/L CdCl_2_ treatments (*p* > 0.05) but increased sharply under the 2 mmol/L CdCl_2_ treatment (Fig. 4C). The trend for APX activity was the same as that for SOD activity (Fig. 4D).

### Gene expression patterns respond to Cd stress in *E. canadensis*

#### Transcriptome sequencing and assembly

To decipher the molecular mechanisms underlying heavy metal tolerance in *E. canadensis,* a comparative transcriptome analysis was carried out to measure the gene expression levels of the control (CK) and 0.5 mmol/L Cd^2+^-treated plants (Cd). A total of 12 cDNA libraries (six root and six shoot libraries) were constructed. After quality evaluation (removing adapters and low-quality reads containing poly-N), a total of 89.51 G clean reads were obtained. These reads were used to construct the reference genome of *E. canadensis*, comprising 122,685 unigenes (corresponding to 229,465 transcripts) with an average length of 785 bp. Among these unigenes, 56,816 (46.31%) were annotated against six public databases (NR, Swiss-Prot, Pfam, COG (Clusters of Orthologous Groups of proteins), GO (Gene Ontology), and KEGG (Kyoto Encyclopedia of Genes and Genomes)). The details of this reference genome are summarized in Table S2.

#### Differentially expressed genes (DEGs) between control and Cd-treated *E. canadensis* plants

**Fig. 5:**
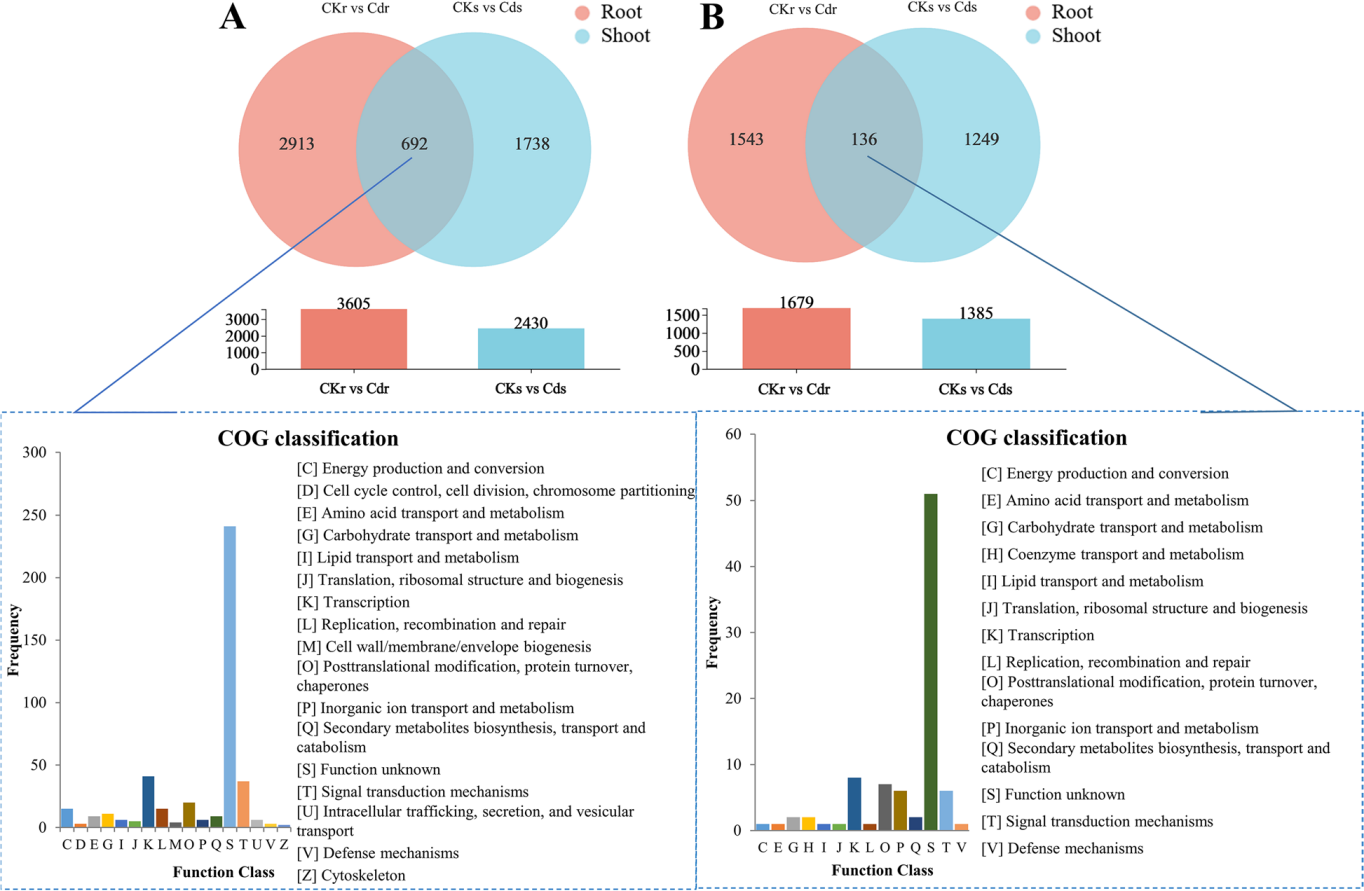
Venn diagram and COG classifications of DEGs in the comparisons of CK *vs.* Cds and CK *vs.* Cdr. **A**: COG classifications of upregulated common DEGs. **B**: COG classifications of downregulated common DEGs. The x-axis represents the COG functional classification (represented by capital letters A-Z; on the right side of the bar chart is the functional description), and the y-axis represents the number of unigenes with such functions

In total, 5,284 (3,605 upregulated and 1,679 downregulated) genes (Fig. 5) were found to be differentially expressed in Cd-stressed roots compared to control roots (CKr *vs.* Cdr); however, only 3,815 (2430 upregulated and 1385 downregulated) DEGs were found in shoots in the comparison of Cd-stressed and control plants (CKs *vs.* Cds), which was significantly lower than the number of DEG in roots (χ^2^ test, p < 0.01), which indicated a transcriptional discrepancy between root and shoot tissue under Cd stress. The number of upregulated DEGs was significantly higher than that of downregulated DEGs in both comparisons (χ^2^ test, *p* < 0.01). In particular, 692 upregulated (Fig. 5A) and 136 downregulated (Fig. 5B) DEGs were identified in both comparisons and were defined as common DEGs. After COG classification, the upregulated common DEGs were found to be mainly enriched in “function unknown”, followed by “transcription” and “signal transduction mechanisms”. The downregulated common DEGs were mainly enriched in “function unknown”, followed by “transcription”, “posttranslational modification”, “signal transduction mechanisms”, and “inorganic ion transport and metabolism”. These common DEGs enriched in the “function unknown” category are listed in Table S3; however, they were not further analysed in this study due to the limited information in public databases.

#### GO functions of the DEGs with tenfold changes in abundance

**Fig. 6:**
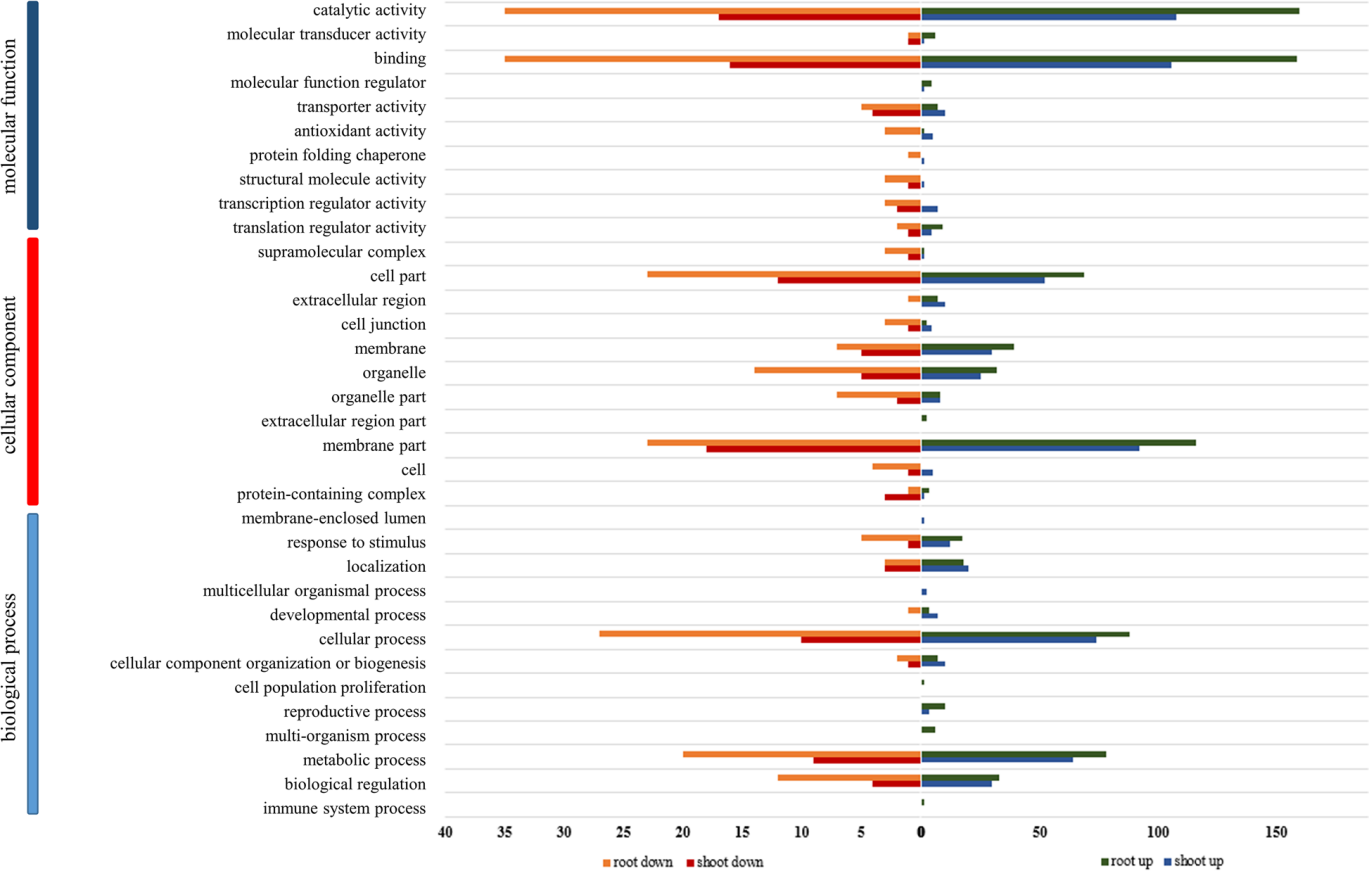
GO assignment of DEGs with tenfold changes in abundance in roots and shoots. DEGs are annotated in three categories: cellular component, molecular function, and biological process. The left and right of the x-axis represent the upregulated (blue for roots and green for shoots) and downregulated genes (red for roots and orange for shoots)

To reduce background interference, the DEGs with tenfold changes in abundance were selected to identify the major functional categories. In total, 402 upregulated (74.86%) and 135 (25.14%) downregulated DEGs in roots and 269 (82.01%) upregulated and 59 (17.99%) downregulated DEGs in shoots were identified. These DEGs were classified into 38 GO terms at level 2 using a hypergeometric distribution (Fig. 6; Table S3). The upregulated DEGs were mainly involved in “catalytic activity”, “binding”, “membrane part”, and “cellular process” in roots. A similar result was observed for upregulated DEGs in shoots. The downregulated DEGs in roots were mainly enriched in “catalytic activity”, “binding”, “cellular process”, “membrane part”, and “cell part”, whereas the downregulated DEGs in shoots were mainly involved in “membrane part”, “catalytic activity”, “binding”, and “cell part”.

#### DEGs enriched in KEGG pathways

A total of 1132 DEGs (including 734 upregulated and 398 downregulated DEGs) in CKs *vs.* Cds and 1349 DEGs (including 937 upregulated and 412 downregulated DEGs) in CKr *vs.* Cdr were annotated to 111 KEGG pathways. The upregulated DEGs in CKs *vs.* Cds were mainly involved in “fatty acid elongation”, “phenylpropanoid biosynthesis”, “flavonoid biosynthesis”, “plant hormone signal transduction”, and “sesquiterpenoid and triterpenoid biosynthesis” (Fig. S1A). The downregulated DEGs in CKs *vs.* Cds were mainly involved in the “photosynthesis”, “photosynthesis-antenna proteins”, “alpha-linolenic acid metabolism”, “linoleic acid metabolism”, and “carbon fixation in photosynthetic organisms” pathways (Fig. S1B). The top 5 enriched KEGG pathways for upregulated DEGs in CKr *vs.* Cdr were “plant‒pathogen interaction”, “MAPK signalling pathway-plant”, “phenylpropanoid biosynthesis”, “sesquiterpenoid and triterpenoid biosynthesis”, and “nitrogen metabolism” (Fig. S1C). The main downregulated DEGs in CKr *vs.* Cdr were enriched in the “plant hormone signal transduction”, “pentose and glucuronate interconversions”, and “zeatin biosynthesis” pathways.

#### KEGG pathways of common DEGs

**Fig. 7:**
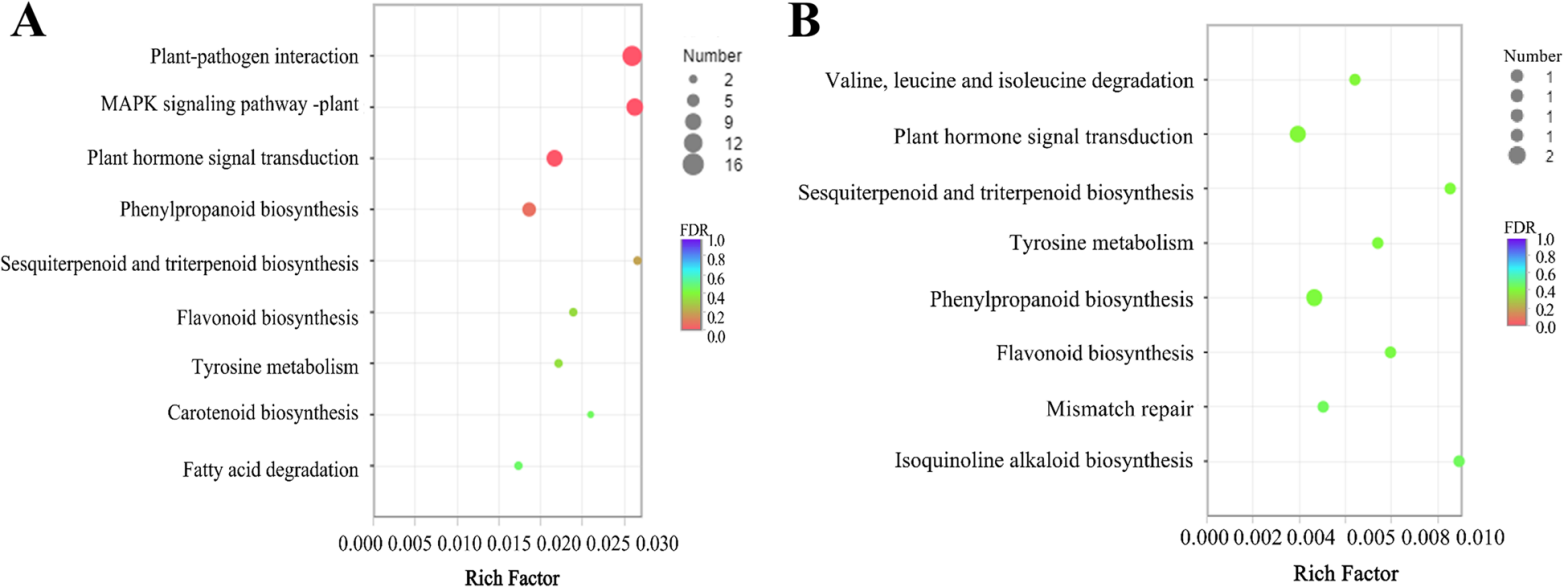
Common DEGs enriched in KEGG pathways. **A**: Upregulated common DEGs involved in KEGG pathways. **B**: Downregulated common DEGs involved in KEGG pathways. The x-axis represents the ratio of the number of unigenes (sample number) enriched (based on the Rich factor) in a pathway to the number of annotated unigenes (background number), and the y-axis represents the pathway name

The common downregulated DEGs were involved in eight KEGG pathways, with only 1 to 2 genes each. These pathways were “valine, leucine and isoleucine degradation” (1 DEG), “plant hormone signal transduction” (2 DEGs), “sesquiterpenoid and triterpenoid biosynthesis” (1 DEG), “tyrosine metabolism” (1 DEG), “phenylpropanoid biosynthesis” (2 DEGs), “flavonoid biosynthesis” (1 DEG), “mismatch repair” (1 DEG), and “isoquinoline alkaloid biosynthesis” (1 DEG) (Fig. 7A). The common upregulated DEGs were mainly involved in “plant‒pathogen interaction” (16 DEGs), “MAPK signalling pathway-plant” (12 DEGs), “plant hormone signal transduction” (11 DEGs), and “phenylpropanoid biosynthesis” (8 DEGs) (Fig. 7B), indicating that these pathways responded to Cd stress.

### Plant hormone signalling pathway response to Cd stress

**Fig. 8:**
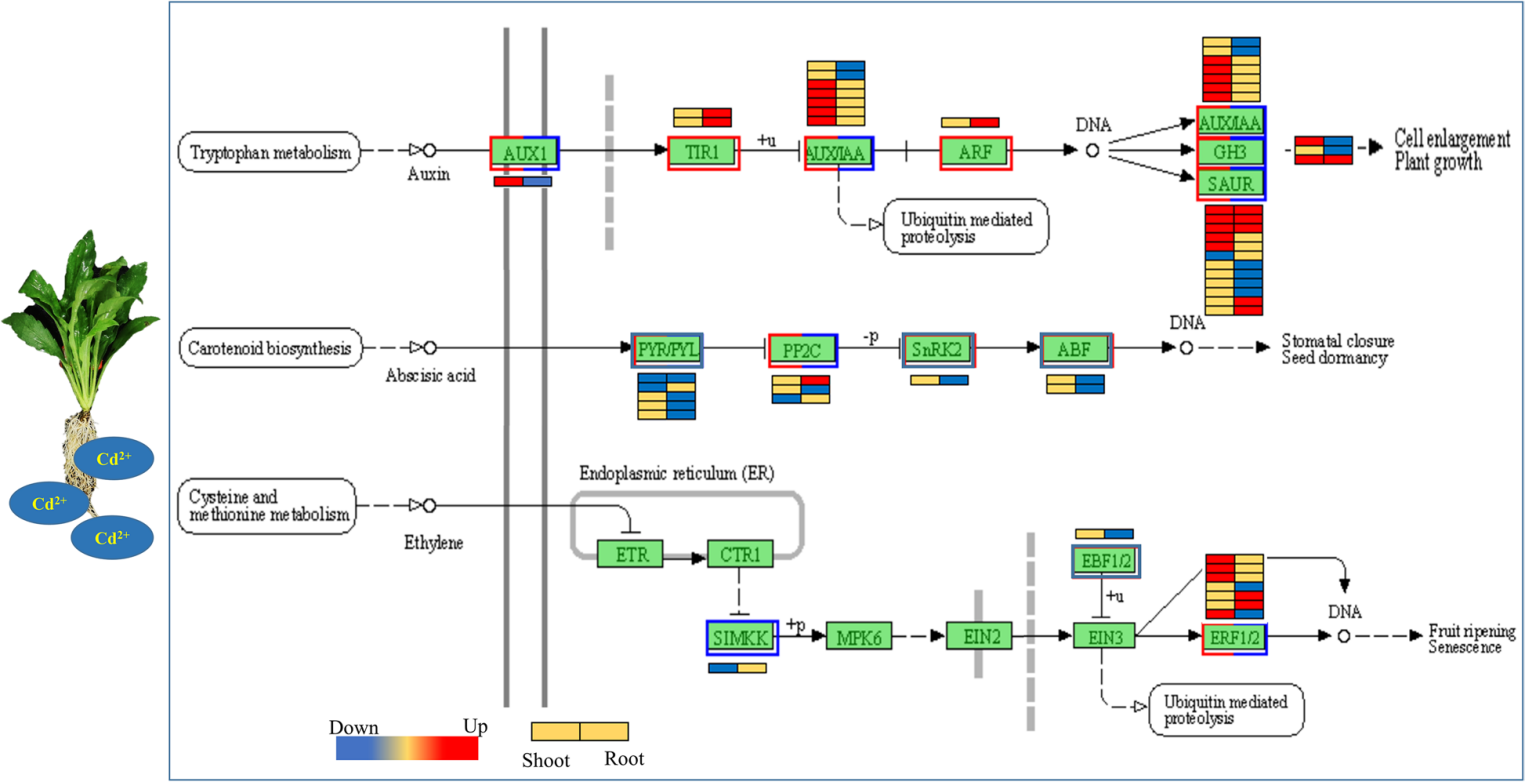
Transcriptional changes in genes involved in plant hormone biosynthesis and plant hormone signal transduction [40–42] in shoots and roots. The expression heatmaps from left to right are for the shoots and roots

We noticed that the plant hormone signalling pathway was significantly enriched in both shoots (53 DEGs) and roots (128 DEGs) under Cd stress. Moreover, both up- and downregulated common DEGs were mainly involved in this pathway (Fig. 8; Table S4). Among them, the gene involved in Trp-auxin (tryptophan-auxin) IAA hormone biosynthesis was upregulated in shoots and downregulated in roots. In the root samples, 18, 9, and 5 DEGs were shown to be involved in IAA, ABA, and ETH signal transduction, respectively. In the shoot samples, 14, 3, and 5 DEGs were shown to be involved in IAA, ABA, and ETH signal transduction, respectively. The three types of early auxin-responsive gene families in IAA signal transduction include Aux/IAA (Aux/indole-3-acetic acid), GH3 (Gretchen Hagen 3), and SAUR (small auxin-up RNA). The gene-encoding proteins Aux/IAA, GH3, and SAUR were mainly (93%) upregulated in the shoots. In the shoots, genes encoding the protein Aux/IAA were also upregulated. Comparatively, in the roots, genes encoding the protein Aux/IAA were downregulated. However, SAUR exhibited a complex expression model. Five SAUR genes were upregulated, and four were downregulated. There was a common DEG encoding the protein PYR/PYL (pyrabatin resistance/pyrabatin resistance 1-like), which is an ABA receptor of the signalling complex involved in ABA signal transduction, that was downregulated under Cd stress in the shoots and roots. The genes encoding the proteins PYR/PYL, SnRK2 (sucrose non-fermenting 1-related protein kinase 2) and ABF (ABA-response element) were downregulated under Cd stress in *E. canadensis.* Only one DEG-encoded protein, PP2C (type 2C protein phosphatase), was upregulated in the roots. In ETH signal transduction, the DEG downregulated in shoots encoded a salt-stress-inducible MAPKK (SIMKK). The DEGs encoding EBF1/2 (ethylene-responsive factor ½) were downregulated in the roots. The different expression levels of IAA, ETH biosynthesis, and signal transduction-related genes between the two different tissues might have resulted in lower Cd content in shoots than in roots.

### Cell wall biosynthesis, antioxidant enzymes, and transcription factor activity in response to Cd stress

**Fig. 9:**
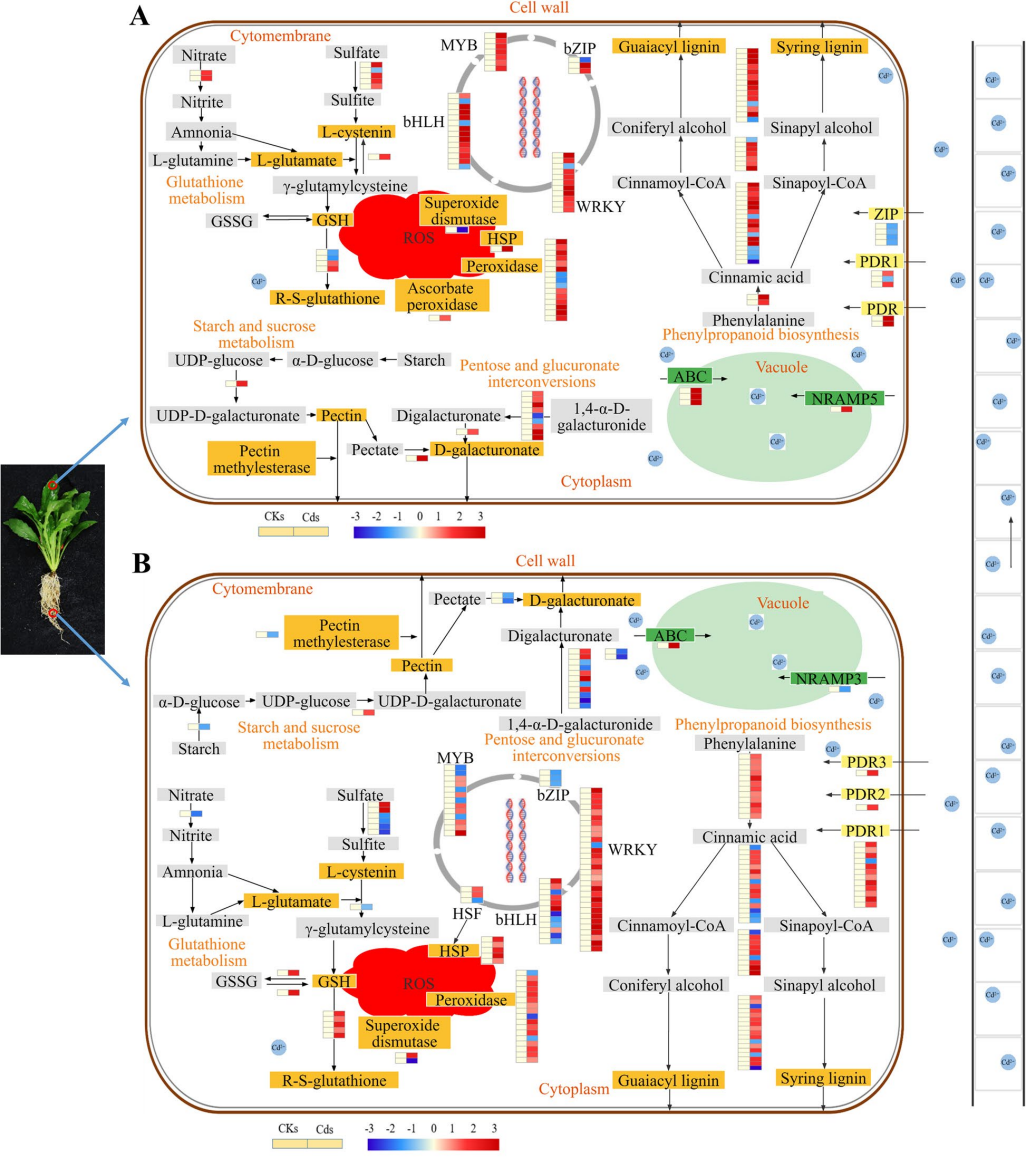
Comparative transcriptomic network in shoots and roots [40–42]. The expression heatmaps from left to right show the CKs and Cds (**A**) and CKr and Cdr (**B**) comparisons

Additionally, the DEGs associated with cell wall biosynthesis, antioxidant enzymes, and transcription factors were also annotated using KEGG pathway analysis (Fig. 9; Table S5). The phenylpropanoid biosynthesis pathway was related to the cell wall; therefore, we analysed two other pathways (pentose and glucuronate interconversion and starch and sucrose metabolism) that were also associated with the cell wall (Fig. 9A). The DEGs involved in pathways related to D-galacturonate synthesis were mainly (80.00%) upregulated in the shoots, and 61.54% were downregulated in the roots. Most (78.05%) of the DEGs associated with guaiacyl lignin and syringyl lignin biosynthesis were upregulated in the shoots, and 77.78% were upregulated in the roots.

There were 17 DEGs (1 gene encoding superoxide dismutase, 1 gene encoding ascorbate peroxidase and 15 genes encoding peroxidases) identified in the shoots in response to ROS detoxification in *E. canadensis* shoots under Cd stress (Fig. 9A). Under Cd stress, the expression levels of four genes were downregulated, and 13 genes were upregulated. In addition, 17 genes encoding peroxidases (3 DEGs were downregulated) and 2 genes encoding superoxide dismutase (1 DEG was downregulated) were detected in the roots when *E. canadensis* was exposed to Cd. One and 5 upregulated DEGs in the shoots and roots were found to be involved in HSP.

The PDR-type ABC transporters (PDR1, PDR2, PDR3) showed distinctly increased expression levels in roots under Cd treatment. However, PDR-type ABC transporters (PDR, PDR1) were more highly expressed in the shoots under Cd treatment. The DEGs associated with the PDR family identified in this study were mostly upregulated (80% in the shoots, 92.85% in the roots). Moreover, compared to the control, the DEGs related to several transcription factor families (WRKY, bHLH, MYB, and bZIP) were more abundant under cadmium treatment. These WRKY genes were consistently upregulated in response to Cd tolerance, except for one downregulated DEG. The bHLH family included 3 upregulated DEGs and 11 downregulated DEGs in the shoots under Cd stress. The bHLH family included 12 DEGs in the roots, of which 6 DEGs were upregulated under Cd stress. The DEGs of the MYB family were upregulated in the Cd-stressed shoots, whereas eight genes were upregulated and five were downregulated in the Cd-stressed roots. The 3 DEGs downregulated in the roots or 3 DEGs (2 genes upregulated, 1 gene downregulated) identified in the shoots were grouped into the bZIP family.

### Validation of the DEGs by qRT‒PCR analysis

To verify the reliability of RNA-seq, 10 DEGs (five upregulated and five downregulated) were randomly chosen for qRT‒PCR analysis. The selected unigene and primer sequences are listed in Table S6. The results showed that the expression levels measured by qRT‒PCR were consistent with the transcriptomic data (Figure S2), supporting the RNA-seq results.

## Discussion

*E. canadensis*, with strong adaptation, large biomass, and high seed abundance, has been recognized as an invasive species in China [36, 37]. In this study, the *E. canadensis* seedlings gathered from a mining area did not show obvious phenotypic changes after treatment with high concentrations of Cd for 7 days (Fig. 1), indicating that *E. canadensis* has strong tolerance to Cd stress. Moreover, the Cd content in shoots was very close to the critical value for Cd hyperaccumulators (100 mg/kg) under the 0.5 mmol/L CdCl_2_ treatment (99.11 ± 3.93 mg/kg) and sharply increased under the 1 mmol/L CdCl_2_ treatment (243.83 ± 5.00 mg/kg) and 2 mmol/L CdCl_2_ treatment (317.57 ± 14.85 mg/kg). Our results suggest that *E. canadensis* can be used as a potential accumulator of Cd. Previously, several studies have also demonstrated that *E. canadensis* has the ability to accumulate Cd [36–38]. These plants, defined as hyperaccumulators of heavy metals, should also have a translocation factor and enrichment factor with values > 1 [43, 44]. In the present study, the enrichment factor in *E. canadensis* ranged from 1.41 to 2.17 under different Cd treatments. However, the translocation factor did not exceed 1.0 in any of the Cd treatments (0.36–0.73). This may be because the excessive Cd in the medium prevented the effective transport of Cd in a short period of time (CdCl_2_ treatment was performed for seven days), as also observed in *Abelmoschus manihot* [45]. However, the Cd accumulation ability of *E. canadensis* was only measured under hydroponic conditions in the present study. Therefore, to measure the ability of phytoremediation in polluted soil, a study involving Cd-polluted soils is necessary in the future.

Previous studies have shown that Cd stress can disrupt the absorption and translocation of essential elements [34, 46] and reduce or even dissolve chloroplasts [6, 47], resulting in the overproduction of ROS [31, 48–50]. The increased activities of antioxidant enzymes, such as SOD, CAT, APX, and POD, are thought to play an important metabolic role in the cellular defence tactics against scavenging ROS generated via Cd exposure [16, 50, 51]. The ability to detoxify metals at the physiological level is also a feature of accumulators or hyperaccumulators [51]. The increase in ROS levels under high Cd stress can cause MDA accumulation and oxidative stress, such as an increase in GR activity [49, 52, 53]. In the present study, the MDA content did not vary notably under the 0.5 mmol/L CdCl_2_ treatment but significantly increased under the 1 mmol/L and 2 mmol/L CdCl_2_ treatments (Fig. 3D). A similar result, that is, a rapid increase in GR activity, was observed under the 1 mmol/L and 2 mmol/L CdCl_2_ treatments (Fig. 3F), indicating that *E. canadensis* experienced severe damage under 1 mmol/L CdCl_2_ treatment. Enhancement of POD and SOD activities is considered a defence strategy against excess ROS in plants [16, 49]. The SOD activity increased markedly in shoots treated with different CdCl_2_ solutions (Fig. 4A); intriguingly, the SOD activity under 2 mmol/L CdCl_2_ treatment was slightly lower than that under 1 mmol/L CdCl_2_ treatment. Moreover, compared to that in the control, the POD activity did not show a significant change under the 0.5 mmol/L and 1 mmol/L CdCl_2_ treatments but increased sharply under the 2 mmol/L CdCl_2_ treatment. These results indicate that SOD activity is activated under low-concentration Cd stress and that POD activity mainly responds to intense Cd stress. Similar results were observed in *B. juncea* under a gradient of Cd treatments [16]. Generally, chloroplasts are highly vulnerable to heavy metal stress because excess heavy metals can dissolve the thylakoid membranes of chloroplasts and hamper enzyme activity for chlorophyll photosynthesis [47, 53]. In our study, the chlorophyll content decreased, but not always significantly, under the different Cd treatments (Fig. 3A-C). Moreover, the chlorophyll content, particularly that of chlorophyll a, did not further decrease with increasing Cd concentrations, further indicating the tolerance of *E. canadensis* to intense Cd stress. Moreover, the encoding gene was significantly upregulated (76.47%) in the shoots when the plant was exposed to 0.5 mmol Cd (Fig. 9A). Additionally, the DEGs were significantly enriched in the catalytic activity term, suggesting that more antioxidants were generated to scavenge intracellular ROS and protect cells against heavy metal stress.

It is believed that hormones influence plant growth and act as indicators of metal toxicity [54, 55]. Several studies have found that excess Cd can decrease the endogenous IAA content but enhance the ABA and ETH levels in plants [55–57]. We noticed that both up- and downregulated common DEGs were significantly enriched in the plant hormone signal transduction pathway (Fig. 7), indicating that these genes encoding hormones likely play an important role in the tolerance of Cd in *E. canadensis.* In the shoots, the expression of genes encoding Aux/IAA, GH3, and SAUR was predominantly upregulated (more 92%) under Cd stress (Fig. 8). However, under Cd stress, auxin-responsive factors (ARFs) were upregulated in the roots. Intracellularly, hormones promote the degradation of the AUX/IAA transcriptional repressor, which is associated with ARFs. This in turn inhibits the transcription of auxin-responsive genes and ultimately affects the intensity of IAA signalling but not its localization. In Cd-exposed *E. canadensis*, IAA localization reduced AUX1 signalling. This result is consistent with the findings of Vanneste and Friml [58]. It has been reported that ABA accumulates rapidly in roots exposed to osmotic stress [59], and then PYR/PYL inactivates the activities of PP2Cs, permitting protein phosphorylation and subsequently activating SnRK2 protein kinases, which thereby activate their downstream targets, ABF/AREB/ABI5 [60]. However, in this study, DEGs involved in PYR/PYL, SnRK2 and ABF were downregulated in the roots. Moreover, two DEGs belonging to the PP2C family were distinctly regulated. The results showed that cadmium stress and the combined interactions between receptors and the core ABA signalling pathway (involving PP2C-SnRK2-ABF [61]) enable a tunable response to stress signalling.

In addition, *E. canadensis* can initiate multiple pathways to counteract the damaging effects. Moreover, in the molecular function category, the catalytic activity term was abundant, suggesting that this organism can produce antioxidant at high levels to scavenge intracellular ROS and protect cells against heavy metal stress. The antioxidant defence system attenuates the damage to *E. canadensis* caused by H_2_O_2_ and lipid peroxidation [62]. The encoding gene was upregulated (76.47%) in the shoots when the plant was exposed to 0.5 mM Cd treatment (Fig. 9A). Heat shock proteins (HSPs) ensure cellular tolerance to stressors, such as oxidative stress [63]. Six HSP DEGs (1 DEG in the shoots and 5 DEGs in the roots) that were upregulated in *E. canadensis* were identified (Fig. 9). These results suggest that *E. canadensis* could also resist oxidative stress. WRKY TFs have been implicated in the defence response of plants [64, 65]. One of the WRKY TFs was downregulated (TRINITY_DN2439_c0_g1), and its predicted NR annotation was WRKY transcription factor 58 (*Helianthus annuus*). Wang et al. [66] showed that WRKY58 plays a negative regulatory role in defence in Arabidopsis. WRKY6 plays an instrumental role in plant pathogen defence, arsenate resistance, and phosphate transport [67]. Recent reports suggest that the AtWRKY41 protein controls seed dormancy by directly regulating the expression of ABI3 [68]. Sun et al. [69] showed that activation of AtWRKY53 expression has a negative regulatory effect on drought resistance. In Banerjee's study, the expression levels of WRKY7, WRKY40, WRKY50, and WRKY57 were upregulated, showing that their role was vital in cold stress tolerance as well as in reducing oxidative stress [70]. Regulation of WRKY70 expression alters resistance to pathogenic bacterial infection in Arabidopsis, suggesting that WRKY70 can control the balance between plant protection and prevention [71]. In this study, the majority of the WRKY transcription factors were upregulated in flycatchers, suggesting that WRKY transcription factors may have a cocoordinated role in Cd tolerance. However, these genes, which showed drastic changes under Cd stress but did not have clear functions, also need to be examined in future studies.

## Materials and methods

### Plant materials and experimental conditions

Seeds were collected from an *E. canadensis* plant grown in a mercury mining area (Danzhai County, China, E107.86°, N26.16°) with a Cd concentration of 0.27 ± 0.03 mg kg^−1^ at pH < 6.0. The seeds were soaked to hasten germination in dishes at 28 °C. When germinated, the seeds were planted in white squares (7 cm in length and width and 10 cm in depth) with vermiculite growing medium and cultivated in a greenhouse under a 16:8 light–dark cycle with a stationary temperature of 22 °C and a relative humidity of 40%. The plants were regularly sprayed with Hoagland nutrient solution until the 12^th^ leaf was generated. Then, plants of uniform size were selected and prepared in 15 × 25 cm white rectangular pots (15 cm in width, 25 cm in length, and 10 cm in depth) in groups of six plants each. The plants were treated with Hoagland nutrient solution with different concentrations of CdCl_2_ (0, 0.5, 1, and 2 mmol/L CdCl_2_) for seven days. The solutions were replaced every three days. At least twelve plants were prepared for each treatment.

### Determination of Cd content

Twelve plants from each treatment were divided into three subsamples, with each treatment providing three replicates. To determine Cd accumulation in shoots and roots, the tested plants were first washed using tap water to remove the residual solution. Then, these samples were divided into above-ground (shoots) and below-ground (roots) fractions. The roots were soaked in 20 mmol/L Na_2_-EDTA for 20 min to remove the adsorbed ions and then rinsed with deionized water for 2 h. Some roots and shoots were frozen in liquid nitrogen and stored at -80 °C for physiological and transcriptome analyses. The remaining material was first dried at 105 °C for 30 min and then dried at 65 °C until completely dehydrated.

These samples were ground to a fine powder, digested in 70% nitric acid solution using poikilothermic treatment (80 °C for 2 h, 120 °C for 2 h, and 160 °C for 4 h), and finally diluted with ultrapure water. The concentrations of Cd in shoots and roots (four replications per treatment) were determined by ICP‒MS (Thermo X Series II).

### Measurements of antioxidant enzyme activities and protein and chlorophyll levels

The measurements of antioxidant enzyme activities in *E. canadensis* shoots from each replicate, including those of superoxide dismutase (SOD), peroxidase (POD), catalase (CAT), and ascorbate peroxidase (APX), were carried out according to the work of Zhang et al. [16] with minor modification. For example, the tested samples were digested using commercial assay kits (Beijing Solarbio Science & Technology Co., Ltd.) according to the manufacturer’s instructions. The activities of SOD, CAT, APX, and POD were determined using a microplate reader (Bio Tek Instrument, Inc., USA) by measuring the absorbance at 560, 240, 290, and 420 nm, respectively. The activity of glutathione reductase (GR) was measured according to the work of Carlberg and Mannervik [72]. The malondialdehyde (MDA) content was determined as described by Yan et al. [31]. To determine the proline content in the samples, a commercial assay kit was used to digest the samples (Beijing Solarbio Science & Technology Co., Ltd.) according to the manufacturer’s instructions. Then, the absorbance of the samples was measured using a microplate reader at 520 nm.

The chlorophyll levels in the samples were determined by the acetone extraction method [73]. Approximately 0.1 g of fresh leaves was ground in liquid nitrogen, and 5 mL of 80% acetone was added. Then, the solution was mixed gently in the dark for 24 h to protect the chlorophyll from light damage. The mixture was centrifuged at 12,000 × g for 15 min. Chlorophyll levels were determined using a microplate reader to measure the absorbance at 663 and 645 nm. Three biological samples per treatment and three technical replicates per sample were used to determine the physiological indices.

### RNA extraction, library preparation, and differentially expressed gene (DEG) determination

The shoots and roots from the control and 0.5 mmol/L CdCl_2_ treatments were collected and immediately stored in liquid nitrogen. Approximately 0.1 g of tissue per sample was used to extract total RNA using a commercial RNA extraction kit (EASYspin Plant RNA kit, Aidlab, China). Then, a Nanodrop 2000 was used to determine the RNA concentration. Both agarose gel electrophoresis (AGE) and RIN values determined by an Agilent 2100 instrument were used to measure RNA integrity. A total of 1.5 µg of qualified RNA (no smear in the AGE gel and RIN values ≥ 8.0) was used to construct a cDNA library following the TruSeq RNA Sample Prep v2 protocol (Illumina, USA). The libraries were sequenced using the Illumina Novaseq 6000 platform to generate 150-bp paired-end reads. Three biological replicates for both the control and the treatment were prepared for sequencing. To obtain clean reads, Trimmomatic version 0.33 [74] was employed to remove adapters and low-quality reads (N of reads < 10% and length of reads < 30 bp) using the following parameters: LEADING: 3, TRAILING: 3, SLIDINGWINDOW: 4:15, MINLEN: 36, LEADING: 3, TRAILING: 3, SLIDINGWINDOW: 4:15, MINLEN: 36, TOPHRED: 33. Then, these qualified reads were de novo assembled into the reference genome using Trinity -2.1.0 with default parameters [75]. Subsequently, the TransRate and CD-HIT packages in R (v. 3.4.3) were used to optimize the reference according to default parameters [76]. All the assembled genes from this reference genome were blasted against six databases, namely, NR, Swiss-Prot, Pfam, COG, GO, and KEGG, to decipher their potential functions using NCBI BLAST 2.2.28^+^ with default parameters. Plant transcription factors were selected after blasting against the PlantTFDB 4.0 database (http://planttfdb.cbi.pku.edu.cn/). The fragments per kilobase of exon per million fragments mapped (FPKM) value was used to assess gene expression levels using RSEM. Differentially expressed genes (DEGs) between the control and treatment groups were adjusted using Benjamini and Hochberg’s approach with a cut-off of *P* < 0.05 and fold changes > 2. For GO annotations of DEGs, GOatools (https://github.com/tanghaibao/GOatools) was used to determine significant enrichment when a corrected P value was less than 0.05.

### Quantitative PCR (qPCR) and statistical analysis

To verify the reliability of RNA-seq, the gene expression levels of the RNA samples were also determined by qRT‒PCR. Ten DEGs, including five upregulated and five downregulated genes, were randomly chosen for qRT‒PCR validation. The primers (Table S6) were designed by utilizing NCBI Primer-Blast. First-strand cDNA synthesis was performed using a HiFiScript gDNA Removal cDNA Synthesis Kit. The *actin* gene was used as an internal housekeeping gene control. A 10 μl qRT‒PCR mixture was prepared by following the protocol for the SYBR-GREEN fluorescent reagents (TIANGEN Biotech, Beijing, China): 5 μl of 2 × SYBR® Premix Ex Taq II, 0.3 μl each of the forward and reverse primers, 1 μl of cDNA, and 3.2 μl of RNase-Free ddH_2_O. The qPCR thermal cycling profile consisted of 95 °C for 3 min, followed by 40 cycles of 95 °C for 5 s and 60 °C for 15 s.

## Availability of data and materials

All data analysed in this study have been deposited in the National Center for Biotechnology Information (NCBI) database under the submission number PRJNA785126. These data are available at https://www.ncbi.nlm.nih.gov/sra/PRJNA785126. 

## Conclusion

In this study, integrative physiological and transcriptome analyses were conducted to demonstrate that *E. canadensis,* with its wide adaptability and large biomass, can be used for Cd phytoremediation. Additionally, the results of the comparative transcriptome analysis indicate that multiple and complex biological processes, particularly the plant hormone signalling pathway, antioxidant enzyme activity, and transcription factor activity, are involved in the Cd tolerance of *E. canadensis*. This study lays the foundation for the phytoremediation of Cd-polluted soils through plant engineering and further provides important resources for phytoremediation.

## Supplementary Information

**Additional file 1:** **Table S1.** Cd content in the roots and shoots of control and treated *E. canadensis* plants.

**Additional file 2:** **Table S2.** Summary of the *E. canadensis* transcriptome assembly.

**Additional file 3:** **Table S3.** GO assignment of DEGs annotated with tenfold changes in abundance in the roots and shoots.

**Additional file 4:** **Figure S1.** Top 10 enriched KEGG pathways of the up- and downregulated DEGs in CKs *vs.* Cds and CKr *vs.* Cdr. The x-axis represents the ratio of DEGs enriched (based on the Rich factor) in a pathway to the annotated unigene number (background number), and the y-axis represents the pathway name.

**Additional file 5:** **Table S4.** DEGs involved in plant hormone biosynthesis and plant hormone signal transduction.

**Additional file 6:** **Table S5.** Cell wall biosynthesis, antioxidant enzyme, and transcription factor activity.

**Additional file 7:** **Table S6.** Primers used for gene expression measurement by qPCR.

**Additional file 8:** **Figure S2.** qRT‒PCR verification of the expression levels of 10 DEGs (five upregulated and four downregulated) determined by RNA-seq. The left y-axis represents the relative expression level, and the right y-axis represents the RNA-seq data (FPKM value). The box with error bars indicates the qRT‒PCR result, and the oblique line represents the FPKM value of gene expression.
