# Supplementary material for: Integrative physiological and transcriptome analyses provide insights into the Cadmium (Cd) tolerance of a Cd accumulator: Erigeron canadensis
Source: BMC Genomics. 2022 Nov 28;23:778. doi: 10.1186/s12864-022-09022-5 (PMC9703714; doi:10.1186/s12864-022-09022-5)
Supplement: Supplementary file 1 — Additional file 1: Table S1. Cd content in the roots and shoots of control and treated E. canadensis plants. [file 12864_2022_9022_MOESM1_ESM.doc]

**Table S1** The Cd content in roots and shoots of controlled and treated Erigeron canadensis plants.

| **Treatment level (mmol L^−1^)** | **Cd in plant tissues (mg kg^−1^)** | | **TF** |
| --- | --- | --- | --- |
|  | **Shoot** | **Root** |  |
| Control | 0.90±0.04d | 1.85±0.10d | ­- |
| 0.5 | 99.12±3.93c | 178.72±6.23c | 0.55±0.02b |
| 1 | 243.83±5.00b | 335.55±8.80b | 0.73±0.02a |
| 2 | 317.57±14.85a | 891.80±32.86a | 0.36±0.02c |
