# Supplementary material for: Integrative physiological and transcriptome analyses provide insights into the Cadmium (Cd) tolerance of a Cd accumulator: Erigeron canadensis
Source: BMC Genomics. 2022 Nov 28;23:778. doi: 10.1186/s12864-022-09022-5 (PMC9703714; doi:10.1186/s12864-022-09022-5)
Supplement: Supplementary file 2 — Additional file 2: Table S2. Summary of the E. canadensis transcriptome assembly. [file 12864_2022_9022_MOESM2_ESM.doc]

**Table S2** Summary Erigeron canadensis of the transcriptome assembly

| **Items** | **Number** |
| --- | --- |
| Total transcripts | 229465 |
| Total bases | 223296012bp |
| Max length | 15602bp |
| Average length | 973.12bp |
| Min length | 201bp |
| N50 | 1497bp |
| Total unigenes | 123328 |
| Total bases | 96825897bp |
| Max length | 15602bp |
| Average length | 785.11bp |
| Min length | 201bp |
| N50 | 1251bp |
