# Supplementary material for: Integrative physiological and transcriptome analyses provide insights into the Cadmium (Cd) tolerance of a Cd accumulator: Erigeron canadensis
Source: BMC Genomics. 2022 Nov 28;23:778. doi: 10.1186/s12864-022-09022-5 (PMC9703714; doi:10.1186/s12864-022-09022-5)
Supplement: Supplementary file 3 — Additional file 3: Table S3. GO assignment of DEGs annotated with tenfold changes in abundance in the roots and shoots. [file 12864_2022_9022_MOESM3_ESM.doc]

**Table S3** GO assignment of ten-fold changes annotated DEGs in root and shoot

| **GO ID** | **Term description** | **Number of upregulated DEGs** | | **Number of downregulated DEGs** | |
| --- | --- | --- | --- | --- | --- |
|  |  | **CKs *vs.* Cds** | **CKr *vs.* Cdr** | **CKs *vs.* Cds** | **CKr *vs.* Cdr** |
| GO:0002376 | immune system process | 0 | 1 | 0 | 0 |
| GO:0065007 | biological regulation | 30 | 33 | 4 | 12 |
| GO:0008152 | metabolic process | 64 | 78 | 9 | 20 |
| GO:0051704 | multi-organism process | 0 | 6 | 0 | 0 |
| GO:0022414 | reproductive process | 3 | 10 | 0 | 0 |
| GO:0008283 | cell population proliferation | 0 | 1 | 0 | 0 |
| GO:0071840 | cellular component organization or biogenesis | 10 | 7 | 1 | 2 |
| GO:0009987 | cellular process | 74 | 88 | 10 | 27 |
| GO:0032502 | developmental process | 7 | 3 | 0 | 1 |
| GO:0032501 | multicellular organismal process | 2 | 0 | 0 | 0 |
| GO:0051179 | localization | 20 | 18 | 3 | 3 |
| GO:0050896 | response to stimulus | 12 | 17 | 1 | 5 |
| GO:0031974 | membrane-enclosed lumen | 1 | 0 | 0 | 0 |
| GO:0032991 | protein-containing complex | 1 | 3 | 3 | 1 |
| GO:0005623 | cell | 5 | 0 | 1 | 4 |
| GO:0044425 | membrane part | 92 | 116 | 18 | 23 |
| GO:0044421 | extracellular region part | 0 | 2 | 0 | 0 |
| GO:0044422 | organelle part | 8 | 8 | 2 | 7 |
| GO:0043226 | organelle | 25 | 32 | 5 | 14 |
| GO:0016020 | membrane | 30 | 39 | 5 | 7 |
| GO:0030054 | cell junction | 4 | 2 | 1 | 3 |
| GO:0005576 | extracellular region | 10 | 7 | 0 | 1 |
| GO:0044464 | cell part | 52 | 69 | 12 | 23 |
| GO:0099080 | supramolecular complex | 1 | 1 | 1 | 3 |
| GO:0045182 | translation regulator activity | 4 | 9 | 1 | 2 |
| GO:0140110 | transcription regulator activity | 7 | 0 | 2 | 3 |
| GO:0005198 | structural molecule activity | 1 | 0 | 1 | 3 |
| GO:0044183 | protein folding chaperone | 1 | 0 | 0 | 1 |
| GO:0016209 | antioxidant activity | 5 | 1 | 0 | 3 |
| GO:0005215 | transporter activity | 10 | 7 | 4 | 5 |
| GO:0098772 | molecular function regulator | 1 | 4 | 0 | 0 |
| GO:0005488 | binding | 106 | 159 | 16 | 35 |
| GO:0060089 | molecular transducer activity | 1 | 6 | 1 | 1 |
| GO:0003824 | catalytic activity | 108 | 160 | 17 | 35 |
