# Supplementary material for: Integrative physiological and transcriptome analyses provide insights into the Cadmium (Cd) tolerance of a Cd accumulator: Erigeron canadensis
Source: BMC Genomics. 2022 Nov 28;23:778. doi: 10.1186/s12864-022-09022-5 (PMC9703714; doi:10.1186/s12864-022-09022-5)
Supplement: Supplementary file 4 — Additional file 4: Figure S1. Top 10 enriched KEGG pathways of the up- and downregulated DEGs in CKs vs. Cds and CKr vs. Cdr. The x-axis represents the ratio of DEGs enriched (based on the Rich factor) in a pathway to the annotated unigene number (background number), and the y-axis represents the pathway name. [file 12864_2022_9022_MOESM4_ESM.doc]

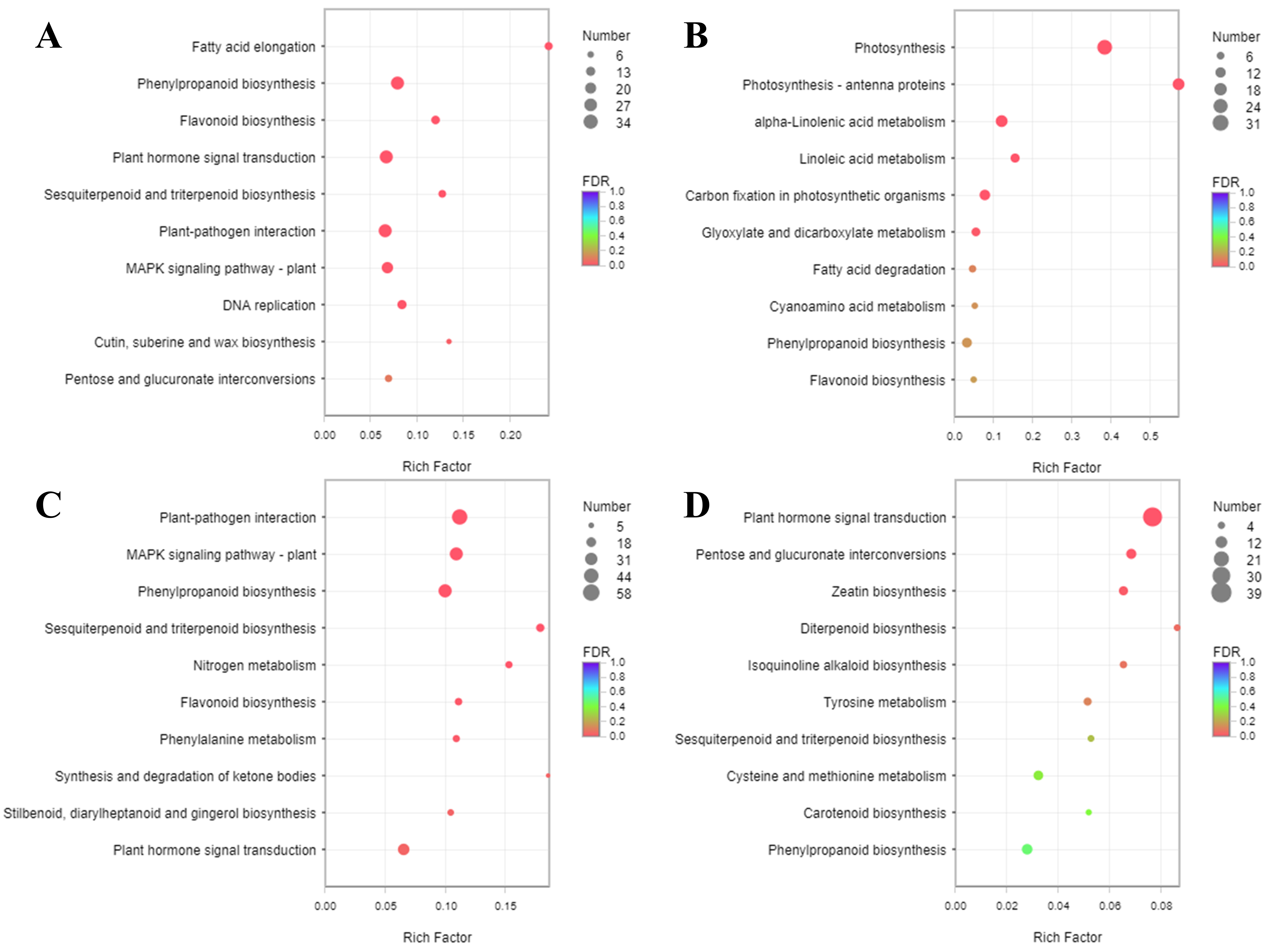


Figuer S1. The top 10 KEGG enrichment pathways of up- and downregulated DEGs in CKs vs. Cds and CKr vs. Cdr. The x-axis represents the ratio of DEGs enriched by the Rich factor in this pathway to the annotated unigene number (background number), and the y-axis represents the pathway name.
