# Supplementary material for: Integrative physiological and transcriptome analyses provide insights into the Cadmium (Cd) tolerance of a Cd accumulator: Erigeron canadensis
Source: BMC Genomics. 2022 Nov 28;23:778. doi: 10.1186/s12864-022-09022-5 (PMC9703714; doi:10.1186/s12864-022-09022-5)
Supplement: Supplementary file 5 — Additional file 5: Table S4. DEGs involved in plant hormone biosynthesis and plant hormone signal transduction. [file 12864_2022_9022_MOESM5_ESM.doc]

**Table S4** DEGs involved in plant hormone biosynthesis and plant hormone signal transduction in plants

| **KEGG** | **ID** | **KO name** | **CKs *vs.* Cds** | **CKr *vs.* Cdr** |
| --- | --- | --- | --- | --- |
|  |  |  |  |  |
| Auxin | TRINITY_DN11767_c0_g1 | AUX1, LAX | up | down |
|  | TRINITY_DN11669_c1_g2 | TIR1 | - | down |
|  | TRINITY_DN11669_c1_g1 | TIR1 | - | down |
|  | TRINITY_DN915_c0_g1 | IAA | up | - |
|  | TRINITY_DN703_c0_g1 | IAA | up | - |
|  | TRINITY_DN4318_c0_g1 | IAA | up | - |
|  | TRINITY_DN5614_c0_g1 | IAA | up | - |
|  | TRINITY_DN46112_c0_g1 | IAA | up | - |
|  | TRINITY_DN13444_c0_g2 | IAA | - | down |
|  | TRINITY_DN11697_c0_g1 | IAA | - | down |
|  | TRINITY_DN43996_c0_g1 | ARF | - | down |
|  | TRINITY_DN5455_c0_g1 | GH3 | up | up |
|  | TRINITY_DN26047_c0_g1 | GH3 | up | down |
|  | TRINITY_DN21627_c0_g1 | GH3 | - | down |
|  | TRINITY_DN14922_c0_g1 | SAUR | down | - |
|  | TRINITY_DN7948_c0_g1 | SAUR | up | - |
|  | TRINITY_DN82823_c0_g1 | SAUR | up | - |
|  | TRINITY_DN7712_c0_g1 | SAUR | up | up |
|  | TRINITY_DN1811_c0_g1 | SAUR | up | - |
|  | TRINITY_DN9010_c0_g1 | SAUR | up | up |
|  | TRINITY_DN22515_c0_g1 | SAUR;ccav | - | down |
|  | TRINITY_DN22569_c0_g1 | SAUR | - | down |
|  | TRINITY_DN23735_c0_g1 | SAUR | - | up |
|  | TRINITY_DN1811_c0_g1 | SAUR | - | up |
|  | TRINITY_DN21875_c0_g2 | SAUR | - | down |
|  | TRINITY_DN5432_c0_g1 | SAUR | - | up |
|  | TRINITY_DN15374_c0_g1 | SAUR | - | down |
| ABA | TRINITY_DN32897_c0_g1 | PYL | down | - |
|  | TRINITY_DN2665_c0_g1 | PYL | down | down |
|  | TRINITY_DN27567_c0_g1 | PYL | - | down |
|  | TRINITY_DN805_c1_g1 | PYL | - | down |
|  | TRINITY_DN4330_c0_g1 | PYL | - | down |
|  | TRINITY_DN2138_c0_g1 | PP2C | down | - |
|  | TRINITY_DN35233_c0_g1 | PP2C | - | down |
|  | TRINITY_DN40710_c2_g1 | PP2C | - | up |
|  | TRINITY_DN27918_c0_g1 | SNRK2 | - | down |
|  | TRINITY_DN11934_c0_g1 | ABF | - | down |
|  | TRINITY_DN8145_c0_g2 | ABF | - | down |
| ETH | TRINITY_DN79538_c0_g2 | MKK4_5 | down | - |
|  | TRINITY_DN19272_c0_g1 | EBF1_2 | - | down |
|  | TRINITY_DN49_c0_g1 | ERF1 | up | - |
|  | TRINITY_DN14495_c0_g1 | ERF1 | up | - |
|  | TRINITY_DN7639_c0_g1 | ERF1 | up | down |
|  | TRINITY_DN12480_c0_g1 | ERF1 | up | - |
|  | TRINITY_DN7068_c0_g1 | ERF1 | - | down |
|  | TRINITY_DN33935_c0_g1 | ERF1 | - | up |
|  | TRINITY_DN53546_c0_g1 | ERF1 | - | up |
