# Supplementary material for: Integrative physiological and transcriptome analyses provide insights into the Cadmium (Cd) tolerance of a Cd accumulator: Erigeron canadensis
Source: BMC Genomics. 2022 Nov 28;23:778. doi: 10.1186/s12864-022-09022-5 (PMC9703714; doi:10.1186/s12864-022-09022-5)
Supplement: Supplementary file 6 — Additional file 6: Table S5. Cell wall biosynthesis, antioxidant enzyme, and transcription factor activity. [file 12864_2022_9022_MOESM6_ESM.doc]

**Table S5-1** Cell wall biosynthesis in root

| **KEGG** | **ID** | **KO name** | **log_2_(fold change)** | |
| --- | --- | --- | --- | --- |
|  |  |  | **CKr** | **Cdr** |
| Glutathione metabolism | TRINITY_DN79038_c0_g1 | GSR, gor | 0.00 | 2.27 |
|  | TRINITY_DN101122_c0_g1 | gpx, btuE, bsaA | 0.00 | 1.97 |
|  | TRINITY_DN86688_c0_g1 | GST, gst | 0.00 | 1.83 |
|  | TRINITY_DN7966_c0_g1 | GST, gst | 0.00 | 1.04 |
|  | TRINITY_DN103457_c0_g1 | GST, gst | 0.00 | 2.14 |
|  | TRINITY_DN33997_c0_g2 | GST, gst | 0.00 | 0.96 |
|  | TRINITY_DN56209_c0_g1 | HPGDS | 0.00 | 2.19 |
|  | TRINITY_DN17771_c0_g1 | CPL3_4;gshA | 0.00 | -0.96 |
| Phenypropanoid biosynthesis | TRINITY_DN59580_c0_g2 | PAL | 0.00 | 1.18 |
|  | TRINITY_DN19044_c0_g1 | PAL | 0.00 | 1.02 |
|  | TRINITY_DN3407_c0_g1 | PAL | 0.00 | 1.00 |
|  | TRINITY_DN3407_c0_g2 | PAL | 0.00 | 1.13 |
|  | TRINITY_DN3407_c3_g1 | PAL | 0.00 | 2.39 |
|  | TRINITY_DN3407_c3_g2 | PAL | 0.00 | 1.24 |
|  | TRINITY_DN10352_c0_g3 | PAL | 0.00 | 1.38 |
|  | TRINITY_DN13299_c1_g1 | PAL | 0.00 | 0.92 |
|  | TRINITY_DN13299_c0_g1 | PAL | 0.00 | 0.98 |
|  | TRINITY_DN13226_c0_g1 | PAL | 0.00 | 1.89 |
|  | TRINITY_DN13226_c0_g2 | PAL | 0.00 | 1.26 |
|  | TRINITY_DN22176_c0_g1 | PAL | 0.00 | 0.95 |
|  | TRINITY_DN22260_c1_g3 | E2.1.1.68, COMT | 0.00 | -1.68 |
|  | TRINITY_DN10472_c0_g4 | E2.1.1.104 | 0.00 | 1.39 |
|  | TRINITY_DN10472_c0_g3 | E2.1.1.104 | 0.00 | 1.37 |
|  | TRINITY_DN3284_c0_g1 | E2.1.1.104 | 0.00 | 1.38 |
|  | TRINITY_DN14796_c0_g4 | E2.1.1.104 | 0.00 | 0.82 |
|  | TRINITY_DN14796_c0_g3 | E2.1.1.104 | 0.00 | 2.08 |
|  | TRINITY_DN36389_c0_g1 | E2.1.1.104 | 0.00 | 0.87 |
|  | TRINITY_DN78441_c0_g1 | E2.3.1.133, HCT | 0.00 | -1.56 |
|  | TRINITY_DN54197_c0_g1 | E2.3.1.133, HCT;HHT1 | 0.00 | 1.26 |
|  | TRINITY_DN1998_c1_g1 | E2.3.1.133, HCT | 0.00 | 1.74 |
|  | TRINITY_DN1998_c0_g3 | E2.3.1.133, HCT | 0.00 | 1.44 |
|  | TRINITY_DN726_c0_g1 | E2.3.1.133, HCT | 0.00 | -1.48 |
|  | TRINITY_DN40330_c0_g1 | E2.3.1.133, HCT | 0.00 | 1.05 |
|  | TRINITY_DN33977_c0_g2 | E2.3.1.133, HCT | 0.00 | -2.27 |
|  | TRINITY_DN15564_c0_g3 | E2.3.1.133, HCT | 0.00 | -1.51 |
|  | TRINITY_DN19698_c0_g1 | CSE | 0.00 | -1.24 |
|  | TRINITY_DN20034_c0_g1 | CAD | 0.00 | -2.27 |
|  | TRINITY_DN4060_c0_g1 | K22395 | 0.00 | 1.48 |
|  | TRINITY_DN28105_c0_g1 | K22395 | 0.00 | 1.42 |
|  | TRINITY_DN22681_c0_g1 | K22395 | 0.00 | 1.66 |
|  | TRINITY_DN22681_c0_g2 | K22395 | 0.00 | 1.77 |
|  | TRINITY_DN6079_c0_g1 | K22395 | 0.00 | -1.57 |
|  | TRINITY_DN10851_c0_g1 | K22395 | 0.00 | 1.84 |
|  | TRINITY_DN10851_c0_g2 | K22395 | 0.00 | 3.02 |
|  | TRINITY_DN55168_c0_g1 | K22395 | 0.00 | 4.98 |
|  | TRINITY_DN4195_c0_g1 | E1.11.1.7 | 0.00 | 1.14 |
|  | TRINITY_DN6335_c0_g1 | E1.11.1.7 | 0.00 | 0.84 |
|  | TRINITY_DN31650_c0_g1 | E1.11.1.7 | 0.00 | -2.28 |
|  | TRINITY_DN4908_c0_g1 | E1.11.1.7 | 0.00 | 1.42 |
|  | TRINITY_DN35720_c0_g1 | E1.11.1.7 | 0.00 | 1.07 |
|  | TRINITY_DN42109_c0_g1 | E1.11.1.7 | 0.00 | 1.39 |
|  | TRINITY_DN17738_c0_g1 | E1.11.1.7 | 0.00 | 1.49 |
|  | TRINITY_DN4379_c0_g1 | E1.11.1.7 | 0.00 | 0.87 |
|  | TRINITY_DN15811_c0_g1 | E1.11.1.7 | 0.00 | 1.26 |
|  | TRINITY_DN244_c0_g1 | E1.11.1.7 | 0.00 | 1.47 |
|  | TRINITY_DN9700_c0_g1 | E1.11.1.7 | 0.00 | -1.24 |
|  | TRINITY_DN13000_c0_g1 | E1.11.1.7 | 0.00 | 0.83 |
|  | TRINITY_DN20881_c0_g1 | E1.11.1.7 | 0.00 | 1.56 |
|  | TRINITY_DN26976_c0_g1 | E1.11.1.7 | 0.00 | -1.22 |
|  | TRINITY_DN50881_c0_g1 | E1.11.1.7 | 0.00 | 1.70 |
|  | TRINITY_DN29462_c0_g2 | E1.11.1.7 | 0.00 | 1.72 |
|  | TRINITY_DN69252_c0_g1 | E1.11.1.7 | 0.00 | -10.91 |
| Pectin methylesterase | TRINITY_DN4150_c0_g1 | E3.1.1.11 | 0.00 | 1.51 |
|  | TRINITY_DN22470_c0_g1 | E3.1.1.11 | 0.00 | 1.94 |
|  | TRINITY_DN33119_c0_g1 | E3.1.1.11 | 0.00 | -1.15 |
|  | TRINITY_DN9119_c1_g1 | E3.1.1.11 | 0.00 | -1.77 |
|  | TRINITY_DN52105_c0_g1 | E3.1.1.11 | 0.00 | 1.37 |
|  | TRINITY_DN10869_c1_g1 | E3.1.1.11 | 0.00 | 2.94 |
|  | TRINITY_DN13327_c0_g1 | E3.2.1.15 | 0.00 | -1.39 |
|  | TRINITY_DN25565_c0_g1 | E3.2.1.15 | 0.00 | 1.95 |
|  | TRINITY_DN36050_c1_g1 | E3.2.1.15 | 0.00 | -1.91 |
|  | TRINITY_DN36050_c2_g1 | E3.2.1.15 | 0.00 | -3.89 |
|  | TRINITY_DN36050_c0_g1 | E3.2.1.15 | 0.00 | -1.80 |
|  | TRINITY_DN20230_c0_g1 | E3.2.1.67 | 0.00 | -1.94 |
|  | TRINITY_DN32306_c0_g1 | E3.2.1.67 | 0.00 | -2.44 |
|  | TRINITY_DN6170_c0_g1 | pel | 0.00 | -1.24 |
|  | TRINITY_DN30767_c0_g1 | pel | 0.00 | -1.81 |
| starch and sucrose metabolism | TRINITY_DN40155_c0_g1 | UGDH, ugd | 0.00 | 1.33 |

**Table S5-2** Cell wall biosynthesis in shoot

| **KEGG** | **ID** | **KO name** | **log_2_(fold change)** | |
| --- | --- | --- | --- | --- |
|  |  |  | **CKs** | **Cds** |
| Glutathione metabolism | TRINITY_DN56209_c0_g1 | HPGDS | 0.00 | -1.19 |
|  | TRINITY_DN33997_c0_g2 | GST, gst | 0.00 | 3.18 |
|  | TRINITY_DN103457_c0_g1 | GST, gst | 0.00 | 3.07 |
|  | TRINITY_DN7966_c0_g1 | GST, gst | 0.00 | -0.08 |
|  | TRINITY_DN86688_c0_g1 | GST, gst | 0.00 | 4.54 |
|  | TRINITY_DN6232_c0_g1 | GST, gst | 0.00 | -1.28 |
|  | TRINITY_DN7907_c0_g3 | GST, gst | 0.00 | -1.50 |
|  | TRINITY_DN1755_c0_g1 | GST, gst | 0.00 | 1.17 |
|  | TRINITY_DN17370_c0_g1 | GST, gst | 0.00 | 1.70 |
|  | TRINITY_DN374_c0_g1 | GGCT | 0.00 | 1.56 |
| Phenypropanoid biosynthesis | TRINITY_DN3407_c3_g2 | PAL | 0.00 | 3.08 |
|  | TRINITY_DN10352_c0_g3 | PAL | 0.00 | 1.66 |
|  | TRINITY_DN14587_c0_g2 | 4CL | 0.00 | -1.31 |
|  | TRINITY_DN62089_c0_g1 | 4CL | 0.00 | -5.12 |
|  | TRINITY_DN28846_c0_g2 | E2.1.1.68, COMT | 0.00 | 11.76 |
|  | TRINITY_DN22260_c1_g2 | E2.1.1.68, COMT | 0.00 | 2.01 |
|  | TRINITY_DN62947_c0_g1 | E2.1.1.68, COMT | 0.00 | 1.46 |
|  | TRINITY_DN5931_c1_g1 | CSE | 0.00 | 2.10 |
|  | TRINITY_DN17658_c0_g1 | E2.1.1.104 | 0.00 | 4.83 |
|  | TRINITY_DN17658_c0_g2 | E2.1.1.104 | 0.00 | 3.93 |
|  | TRINITY_DN33781_c0_g1 | E2.1.1.104 | 0.00 | 1.44 |
|  | TRINITY_DN17105_c0_g1 | E2.3.1.133, HCT;HHT1 | 0.00 | -1.13 |
|  | TRINITY_DN1998_c0_g1 | E2.3.1.133, HCT | 0.00 | 2.06 |
|  | TRINITY_DN34059_c0_g2 | E2.3.1.133, HCT | 0.00 | 5.02 |
|  | TRINITY_DN3545_c0_g1 | E2.3.1.133, HCT | 0.00 | 1.32 |
|  | TRINITY_DN14679_c0_g1 | E2.3.1.133, HCT | 0.00 | -0.96 |
|  | TRINITY_DN28513_c0_g1 | E2.3.1.133, HCT | 0.00 | 3.33 |
|  | TRINITY_DN16110_c0_g2 | E2.3.1.133, HCT | 0.00 | 3.24 |
|  | TRINITY_DN21447_c0_g1 | E2.3.1.133, HCT | 0.00 | -1.04 |
|  | TRINITY_DN96478_c0_g1 | E2.3.1.133, HCT | 0.00 | -1.13 |
|  | TRINITY_DN14587_c0_g2 | E2.3.1.133, HCT | 0.00 | -1.31 |
|  | TRINITY_DN62089_c0_g1 | E2.3.1.133, HCT | 0.00 | -5.12 |
|  | TRINITY_DN20034_c0_g1 | CAD | 0.00 | -0.93 |
|  | TRINITY_DN4060_c1_g1 | K22395 | 0.00 | 2.30 |
|  | TRINITY_DN35636_c0_g2 | K22395 | 0.00 | 2.09 |
|  | TRINITY_DN21778_c0_g1 | K22395 | 0.00 | 1.74 |
|  | TRINITY_DN28105_c0_g1 | K22395 | 0.00 | 1.22 |
|  | TRINITY_DN22681_c0_g2 | K22395 | 0.00 | 1.27 |
|  | TRINITY_DN55168_c0_g1 | K22395 | 0.00 | 8.68 |
|  | TRINITY_DN4908_c0_g1 | E1.11.1.7 | 0.00 | 17.93 |
|  | TRINITY_DN6953_c0_g1 | E1.11.1.7 | 0.00 | 3.78 |
|  | TRINITY_DN9825_c0_g1 | E1.11.1.7 | 0.00 | 3.56 |
|  | TRINITY_DN9988_c0_g1 | E1.11.1.7 | 0.00 | 1.43 |
|  | TRINITY_DN23428_c0_g1 | E1.11.1.7 | 0.00 | 2.15 |
|  | TRINITY_DN5769_c1_g1 | E1.11.1.7 | 0.00 | 1.40 |
|  | TRINITY_DN42109_c0_g1 | E1.11.1.7 | 0.00 | 5.16 |
|  | TRINITY_DN2230_c0_g1 | E1.11.1.7 | 0.00 | 2.43 |
|  | TRINITY_DN21377_c0_g1 | E1.11.1.7 | 0.00 | 1.73 |
|  | TRINITY_DN285_c0_g1 | E1.11.1.7 | 0.00 | 1.82 |
|  | TRINITY_DN21466_c0_g1 | E1.11.1.7 | 0.00 | -1.03 |
|  | TRINITY_DN13000_c0_g1 | E1.11.1.7 | 0.00 | 1.08 |
|  | TRINITY_DN13000_c0_g3 | E1.11.1.7 | 0.00 | 4.72 |
|  | TRINITY_DN26976_c0_g1 | E1.11.1.7 | 0.00 | -1.46 |
| Pectin methylesterase | TRINITY_DN4150_c0_g1 | E3.1.1.11 | 0.00 | 1.24 |
|  | TRINITY_DN25712_c0_g1 | E3.1.1.11 | 0.00 | 1.22 |
|  | TRINITY_DN9119_c1_g1 | E3.1.1.11 | 0.00 | 3.33 |
|  | TRINITY_DN5808_c0_g1 | E3.1.1.11 | 0.00 | 1.18 |
|  | TRINITY_DN25159_c0_g3 | E3.1.1.11 | 0.00 | -2.29 |
|  | TRINITY_DN11292_c0_g1 | E3.1.1.11 | 0.00 | -1.20 |
|  | TRINITY_DN102942_c0_g1 | E3.1.1.11 | 0.00 | 1.22 |
|  | TRINITY_DN9652_c0_g1 | E3.1.1.11 | 0.00 | 3.93 |
|  | TRINITY_DN27374_c0_g1 | E3.2.1.15 | 0.00 | 12.03 |
|  | TRINITY_DN14586_c0_g1 | E3.2.1.67 | 0.00 | 1.30 |
|  | TRINITY_DN8945_c0_g1 | pel | 0.00 | 2.78 |
| starch and sucrose metabolism | TRINITY_DN4502_c0_g2 | UGDH, ugd | 0.00 | 1.99 |

**Table S5-3** antioxidant enzyme, and transcription factor activity in root

| **KEGG** | **ID** | **Symbol** | **Description** | **log_2_(fold change)** | |
| --- | --- | --- | --- | --- | --- |
|  |  |  |  | **CKr** | **Cdr** |
| PDR | TRINITY_DN6225_c0_g2 | PDR1 | XP_024972595.1(pleiotropic drug resistance protein 1-like isoform X3 [Cynara cardunculus var. scolymus]) | 0.00 | 1.79 |
|  | TRINITY_DN1042_c0_g1 | PDR1 | XP_021984348.1(pleiotropic drug resistance protein 1-like [Helianthus annuus]) | 0.00 | 0.70 |
|  | TRINITY_DN17642_c0_g1 | PDR1 | XP_023761023.1(pleiotropic drug resistance protein 1-like [Lactuca sativa]) | 0.00 | 1.83 |
|  | TRINITY_DN1042_c4_g1 | PDR1 | XP_022018102.1(pleiotropic drug resistance protein 1-like [Helianthus annuus]) | 0.00 | -1.52 |
|  | TRINITY_DN7350_c0_g1 | PDR1 | XP_022010113.1(pleiotropic drug resistance protein 1-like [Helianthus annuus]) | 0.00 | 1.90 |
|  | TRINITY_DN66659_c2_g1 | PDR1 | XP_022018102.1(pleiotropic drug resistance protein 1-like [Helianthus annuus]) | 0.00 | 1.27 |
|  | TRINITY_DN1865_c0_g1 | PDR1 | XP_022018102.1(pleiotropic drug resistance protein 1-like [Helianthus annuus]) | 0.00 | 0.81 |
|  | TRINITY_DN8292_c0_g1 | PDR1 | XP_024972592.1(pleiotropic drug resistance protein 1-like isoform X1 [Cynara cardunculus var. scolymus]) | 0.00 | 2.32 |
|  | TRINITY_DN18162_c0_g1 | PDR1 | AJK30638.1(pleiotropic drug resistance transporter, partial [Panax ginseng]) | 0.00 | 1.57 |
|  | TRINITY_DN10661_c0_g5 | PDR1 | XP_024969929.1(pleiotropic drug resistance protein 1-like [Cynara cardunculus var. scolymus]) | 0.00 | 0.97 |
|  | TRINITY_DN10661_c0_g4 | PDR1 | XP_024972595.1(pleiotropic drug resistance protein 1-like isoform X3 [Cynara cardunculus var. scolymus]) | 0.00 | 2.37 |
|  | TRINITY_DN27273_c0_g3 | PDR1 | PWA73279.1(pleiotropic drug resistance protein 1 [Artemisia annua]) | 0.00 | 1.32 |
|  | TRINITY_DN10661_c0_g1 | PDR2 | XP_024991917.1(pleiotropic drug resistance protein 2-like isoform X1 [Cynara cardunculus var. scolymus]) | 0.00 | 1.50 |
|  | TRINITY_DN29899_c0_g1 | PDR3 | XP_024966212.1(pleiotropic drug resistance protein 3-like [Cynara cardunculus var. scolymus]) | 0.00 | 1.87 |
| WRKY | TRINITY_DN8_c0_g1 | WRKY41 | XP_021972772.1(probable WRKY transcription factor 41 [Helianthus annuus]) | 0.00 | 2.90 |
|  | TRINITY_DN3356_c0_g1 | WRKY54 | PWA98304.1(WRKY DNA-binding protein 54 [Artemisia annua]) | 0.00 | 1.59 |
|  | TRINITY_DN25581_c0_g1 | WRKY | PWA65019.1(DNA-binding WRKY [Artemisia annua]) | 0.00 | 1.62 |
|  | TRINITY_DN7961_c0_g1 | WRKY30 | XP_024992000.1(probable WRKY transcription factor 30 [Cynara cardunculus var. scolymus]) | 0.00 | 1.62 |
|  | TRINITY_DN8181_c0_g1 | WRKY11 | PWA58192.1(WRKY11 [Artemisia annua]) | 0.00 | 0.91 |
|  | TRINITY_DN17439_c0_g1 | WRKY50 | XP_021981679.1(probable WRKY transcription factor 50 [Helianthus annuus]) | 0.00 | 1.84 |
|  | TRINITY_DN15993_c0_g1 | WRKY7 | XP_024978063.1(probable WRKY transcription factor 7 [Cynara cardunculus var. scolymus]) | 0.00 | 1.37 |
|  | TRINITY_DN1472_c1_g2 | WRKY17 | XP_023740532.1(probable WRKY transcription factor 17 [Lactuca sativa]) | 0.00 | 0.80 |
|  | TRINITY_DN8700_c0_g1 | WRKY26 | XP_024981438.1(probable WRKY transcription factor 26 [Cynara cardunculus var. scolymus]) | 0.00 | 0.79 |
|  | TRINITY_DN526_c0_g1 | WRKY25 | XP_023750044.1(LOW QUALITY PROTEIN: probable WRKY transcription factor 25 [Lactuca sativa]) | 0.00 | 1.67 |
|  | TRINITY_DN2439_c0_g1 | WRKY58 | XP_022011318.1(probable WRKY transcription factor 58 [Helianthus annuus]) | 0.00 | -1.50 |
|  | TRINITY_DN21793_c0_g1 | WRKY40 | XP_024996505.1(probable WRKY transcription factor 40 [Cynara cardunculus var. scolymus]) | 0.00 | 2.43 |
|  | TRINITY_DN14145_c0_g1 | WRKY46 | XP_024996542.1(probable WRKY transcription factor 46 [Cynara cardunculus var. scolymus]) | 0.00 | 2.03 |
|  | TRINITY_DN19133_c0_g1 | WRKY | PWA44385.1(DNA-binding WRKY [Artemisia annua]) | 0.00 | 1.21 |
|  | TRINITY_DN968_c0_g2 | WRKY | AZD51823.1(WRKY family transcription factor [Eschenbachia blinii]) | 0.00 | 2.33 |
|  | TRINITY_DN12061_c0_g1 | WRKY6 | XP_021989466.1(WRKY transcription factor 6-like [Helianthus annuus]) | 0.00 | 0.89 |
|  | TRINITY_DN3197_c0_g2 | WRKY5 | PWA77996.1(WRKY5 [Artemisia annua]) | 0.00 | 0.57 |
|  | TRINITY_DN6820_c0_g1 | WRKY29 | XP_024994816.1(probable WRKY transcription factor 29 [Cynara cardunculus var. scolymus]) | 0.00 | 1.07 |
|  | TRINITY_DN20012_c0_g1 | WRKY75 | XP_023770722.1(probable WRKY transcription factor 75 [Lactuca sativa]) | 0.00 | 2.77 |
|  | TRINITY_DN12421_c2_g1 | WRKY19 | XP_024966118.1(probable WRKY transcription factor 19 [Cynara cardunculus var. scolymus]) | 0.00 | 1.55 |
|  | TRINITY_DN33782_c0_g1 | WRKY40 | XP_022004259.1(probable WRKY transcription factor 40 [Helianthus annuus]) | 0.00 | 3.54 |
|  | TRINITY_DN1725_c0_g1 | WRKY70 | XP_023737392.1(probable WRKY transcription factor 70 [Lactuca sativa]) | 0.00 | 1.98 |
|  | TRINITY_DN3195_c0_g1 | WRKY24 | XP_024974213.1(WRKY transcription factor WRKY24-like [Cynara cardunculus var. scolymus]) | 0.00 | 2.56 |
|  | TRINITY_DN7084_c1_g1 | WRKY41 | XP_023770179.1(probable WRKY transcription factor 41 [Lactuca sativa]) | 0.00 | 2.64 |
|  | TRINITY_DN7084_c1_g3 | WRKY53 | AJF11719.1(WRKY53 [Chrysanthemum x morifolium]) | 0.00 | 2.61 |
|  | TRINITY_DN6955_c0_g1 | WRKY | PWA64644.1(DNA-binding WRKY [Artemisia annua]) | 0.00 | 4.05 |
|  | TRINITY_DN78810_c0_g1 | WRKY50 | XP_024959905.1(probable WRKY transcription factor 50 isoform X2 [Cynara cardunculus var. scolymus]) | 0.00 | 2.51 |
|  | TRINITY_DN2436_c0_g1 | WRKY57 | XP_021998708.1(probable WRKY transcription factor 57 [Helianthus annuus]) | 0.00 | 0.95 |
|  | TRINITY_DN84610_c0_g1 | WRKY34 | XP_024968998.1(probable WRKY transcription factor 34 [Cynara cardunculus var. scolymus]) | 0.00 | 3.51 |
|  | TRINITY_DN30495_c0_g1 | WRKY | PWA68294.1(WRKY domain-containing protein [Artemisia annua]) | 0.00 | 2.41 |
| bHLH | TRINITY_DN17293_c0_g1 | bHLH041 | XP_023756964.1(putative transcription factor bHLH041 [Lactuca sativa]) | 0.00 | 2.47 |
|  | TRINITY_DN6543_c0_g1 | bHLH168 | XP_021995755.1(transcription factor bHLH168 [Helianthus annuus]) | 0.00 | 1.10 |
|  | TRINITY_DN17445_c0_g1 | bHLH96-like | XP_024971007.1(transcription factor bHLH96-like [Cynara cardunculus var. scolymus]) | 0.00 | -1.90 |
|  | TRINITY_DN6801_c0_g1 | bHLH106-like | XP_024975551.1(transcription factor bHLH106-like [Cynara cardunculus var. scolymus]) | 0.00 | 1.39 |
|  | TRINITY_DN24325_c0_g1 | bHLH18-like | XP_023757922.1(transcription factor bHLH18-like isoform X1 [Lactuca sativa]) | 0.00 | 1.90 |
|  | TRINITY_DN35181_c0_g1 | bHLH79 isoform X2 | XP_021983964.1(transcription factor bHLH79 isoform X2 [Helianthus annuus]) | 0.00 | 4.08 |
|  | TRINITY_DN819_c0_g2 | bHLH75-like | XP_024986773.1(transcription factor bHLH75-like [Cynara cardunculus var. scolymus]) | 0.00 | -2.68 |
|  | TRINITY_DN8574_c0_g1 | bHLH143-like | XP_024971872.1(transcription factor bHLH143-like [Cynara cardunculus var. scolymus]) | 0.00 | -1.35 |
|  | TRINITY_DN12602_c0_g1 | bHLH62-like | XP_021978131.1(transcription factor bHLH62-like [Helianthus annuus]) | 0.00 | -1.09 |
|  | TRINITY_DN14049_c0_g1 | bHLH130-like | XP_023729761.1(transcription factor bHLH130-like [Lactuca sativa]) | 0.00 | 0.73 |
|  | TRINITY_DN43833_c0_g1 | bHLH63-like | XP_024979577.1(transcription factor bHLH63-like [Cynara cardunculus var. scolymus]) | 0.00 | -2.33 |
|  | TRINITY_DN2646_c0_g1 | bHLH78-like | XP_021977951.1(transcription factor bHLH78-like [Helianthus annuus]) | 0.00 | -1.15 |
| MYB | TRINITY_DN5925_c0_g1 | MYB59-like isoform X1 | XP_024981431.1(transcription factor MYB59-like isoform X1 [Cynara cardunculus var. scolymus]) | 0.00 | -1.73 |
|  | TRINITY_DN56585_c0_g1 | MYB44-like | XP_022005508.1(transcription factor MYB44-like [Helianthus annuus]) | 0.00 | -1.77 |
|  | TRINITY_DN1738_c0_g2 | MYB44-like | XP_023746650.1(transcription factor MYB44-like [Lactuca sativa]) | 0.00 | 0.80 |
|  | TRINITY_DN1738_c0_g1 | MYB44-like | XP_024977738.1(transcription factor MYB44-like [Cynara cardunculus var. scolymus]) | 0.00 | 0.80 |
|  | TRINITY_DN42641_c0_g1 | MYB78-like | XP_024994195.1(transcription factor MYB78-like [Cynara cardunculus var. scolymus]) | 0.00 | -1.47 |
|  | TRINITY_DN16590_c0_g1 | MYB44-like | XP_021983625.1(transcription factor MYB44-like [Helianthus annuus]) | 0.00 | 1.01 |
|  | TRINITY_DN12694_c0_g1 | MYB13-like | XP_024994309.1(transcription factor MYB13-like [Cynara cardunculus var. scolymus]) | 0.00 | -1.53 |
|  | TRINITY_DN15711_c0_g1 | MYB20-like | XP_023756230.1(transcription factor MYB20-like [Lactuca sativa]) | 0.00 | 1.19 |
|  | TRINITY_DN7588_c0_g1 | MYB14-like | XP_023733503.1(transcription factor MYB14-like [Lactuca sativa]) | 0.00 | 1.09 |
|  | TRINITY_DN25013_c0_g1 | MYB1R1-like | XP_023732114.1(transcription factor MYB1R1-like [Lactuca sativa]) | 0.00 | -1.43 |
|  | TRINITY_DN17295_c0_g1 | MYB10-like | XP_023764613.1(transcription factor MYB10-like [Lactuca sativa]) | 0.00 | 1.12 |
|  | TRINITY_DN365_c0_g1 | MYB30-like | XP_024978056.1(transcription factor MYB30-like [Cynara cardunculus var. scolymus]) | 0.00 | 0.87 |
|  | TRINITY_DN41979_c0_g1 | MYB4-like | XP_024987600.1(transcription factor MYB4-like [Cynara cardunculus var. scolymus]) | 0.00 | 3.34 |
| bZIP | TRINITY_DN8145_c0_g2 | bZIP46-like isoform X2 | XP_022034155.1(bZIP transcription factor 46-like isoform X2 [Helianthus annuus]) | 0.00 | -1.39 |
|  | TRINITY_DN7880_c0_g1 | bZIP11-like | XP_021998431.1(bZIP transcription factor 11-like [Helianthus annuus]) | 0.00 | -1.30 |
|  | TRINITY_DN7880_c1_g1 | bZIP11-like | XP_023769704.1(bZIP transcription factor 11-like [Lactuca sativa]) | 0.00 | -1.21 |
| HSF | TRINITY_DN4667_c0_g1 | HSF | PWA55707.1(Heat shock factor (HSF)-type, DNA-binding [Artemisia annua]) | 0.00 | 1.21 |
|  | TRINITY_DN982_c0_g1 | HSF | PWA77380.1(Heat shock factor (HSF)-type, DNA-binding [Artemisia annua]) | 0.00 | 1.27 |
|  | TRINITY_DN20904_c0_g1 | HSF | PWA74189.1(Heat shock factor (HSF)-type, DNA-binding [Artemisia annua]) | 0.00 | -1.51 |
| peroxidase | TRINITY_DN9700_c0_g1 | heme peroxidase | PWA47367.1(heme peroxidase [Artemisia annua]) | 0.00 | -1.25 |
|  | TRINITY_DN35720_c0_g1 | peroxidase superfamily | PWA97649.1(peroxidase superfamily protein [Artemisia annua]) | 0.00 | 1.05 |
|  | TRINITY_DN50881_c0_g1 | peroxidase | PWA66053.1(peroxidase [Artemisia annua]) | 0.00 | 1.68 |
|  | TRINITY_DN244_c0_g1 | peroxidase N1-like | XP_024963165.1(peroxidase N1-like [Cynara cardunculus var. scolymus]) | 0.00 | 1.46 |
|  | TRINITY_DN29462_c0_g2 | heme peroxidase | PWA95898.1(heme peroxidase [Artemisia annua]) | 0.00 | 1.71 |
|  | TRINITY_DN42109_c0_g1 | peroxidase 72 | XP_023749766.1(peroxidase 72-like [Lactuca sativa]) | 0.00 | 1.37 |
|  | TRINITY_DN6335_c0_g1 | peroxidase 12 | XP_023758012.1(peroxidase 12-like [Lactuca sativa]) | 0.00 | 0.83 |
|  | TRINITY_DN4379_c0_g1 | peroxidase | PWA46198.1(peroxidase [Artemisia annua]) | 0.00 | 0.85 |
|  | TRINITY_DN31650_c0_g1 | peroxidase superfamily | OTF85337.1(putative peroxidase superfamily protein [Helianthus annuus]) | 0.00 | -2.28 |
|  | TRINITY_DN101122_c0_g1 | Glutathione peroxidase | KVH95382.1(Glutathione peroxidase [Cynara cardunculus var. scolymus]) | 0.00 | 1.95 |
|  | TRINITY_DN15811_c0_g1 | peroxidase P7 | XP_021987800.1(peroxidase P7-like [Helianthus annuus]) | 0.00 | 1.24 |
|  | TRINITY_DN20881_c0_g1 | heme peroxidase | OTG10320.1(putative heme peroxidase [Helianthus annuus]) | 0.00 | 1.54 |
|  | TRINITY_DN26976_c0_g1 | peroxidase 17 | XP_021990203.1(peroxidase 17 [Helianthus annuus]) | 0.00 | -1.24 |
|  | TRINITY_DN8267_c0_g1 | heme peroxidase | PWA75013.1(heme peroxidase [Artemisia annua]) | 0.00 | 0.97 |
|  | TRINITY_DN17738_c0_g1 | peroxidase P7 | XP_023771007.1(peroxidase P7 [Lactuca sativa]) | 0.00 | 1.47 |
|  | TRINITY_DN4908_c0_g1 | peroxidase 4 | XP_023731051.1(peroxidase 4-like [Lactuca sativa]) | 0.00 | 1.39 |
|  | TRINITY_DN13000_c0_g1 | basic peroxidase | XP_022018579.1(basic peroxidase-like [Helianthus annuus]) | 0.00 | 0.81 |
| HSP | TRINITY_DN77350_c0_g4 | HSP70-2 | AAR17079.1(heat shock protein 70-2 [Nicotiana tabacum]) | 0.00 | 2.00 |
|  | TRINITY_DN15629_c0_g2 | 17.9 kDa class II HSP | XP_024960503.1(17.9 kDa class II heat shock protein-like [Cynara cardunculus var. scolymus]) | 0.00 | 0.87 |
|  | TRINITY_DN40747_c0_g1 | HSP90 | XP_023878992.1(heat shock protein 90 [Quercus suber]) | 0.00 | 2.02 |
|  | TRINITY_DN23537_c1_g1 | HSP70 | OTG36297.1(putative heat shock protein 70 family [Helianthus annuus]) | 0.00 | 2.50 |
|  | TRINITY_DN8057_c0_g1 | 17.9 kDa class II HSP | XP_023735836.1(17.9 kDa class II heat shock protein-like [Lactuca sativa]) | 0.00 | 0.90 |
| superoxide dismutase | TRINITY_DN77389_c0_g1 | superoxide dismutase | VVA16421.1(PREDICTED: superoxide dismutase [Prunus dulcis]) | 0.00 | 2.06 |
|  | TRINITY_DN46062_c0_g1 | iron superoxide dismutase | APT43084.1(iron superoxide dismutase [Solanum melongena]) | 0.00 | -9.37 |

**Table S5-4** antioxidant enzyme, and transcription factor activity in shoot

| **KEGG** | **ID** | **Symbol** | **Description** | **log_2_(fold change)** | |
| --- | --- | --- | --- | --- | --- |
|  |  |  |  | **CKs** | **Cds** |
| PDR | TRINITY_DN20344_c1_g1 | PDR1 | XP_021984347.1(pleiotropic drug resistance protein 1-like [Helianthus annuus]) | 0.00 | 1.21 |
|  | TRINITY_DN19017_c0_g1 | PDR1 | XP_022000956.1(pleiotropic drug resistance protein 1-like [Helianthus annuus]) | 0.00 | -0.96 |
|  | TRINITY_DN15437_c0_g1 | PDR1 | XP_023759597.1(pleiotropic drug resistance protein 1-like isoform X2 [Lactuca sativa]) | 0.00 | 1.47 |
|  | TRINITY_DN49348_c1_g1 | PDR | OTG16837.1(putative pleiotropic drug resistance protein PDR/CDR [Helianthus annuus]) | 0.00 | 4.12 |
|  | TRINITY_DN23539_c0_g2 | PDR | OTG16837.1(putative pleiotropic drug resistance protein PDR/CDR [Helianthus annuus]) | 0.00 | 3.61 |
| WRKY | TRINITY_DN8_c0_g1 | WRKY41 | XP_021972772.1(probable WRKY transcription factor 41 [Helianthus annuus]) | 0.00 | 3.34 |
|  | TRINITY_DN13646_c0_g1 | WRKY27 isoform X1 | XP_023728918.1(probable WRKY transcription factor 27 isoform X1 [Lactuca sativa]) | 0.00 | 1.97 |
|  | TRINITY_DN165_c0_g1 | WRKY65 isoform X2 | XP_023733038.1(probable WRKY transcription factor 65 isoform X2 [Lactuca sativa]) | 0.00 | -1.05 |
|  | TRINITY_DN968_c0_g2 | WRKY family | AZD51823.1(WRKY family transcription factor [Eschenbachia blinii]) | 0.00 | 1.81 |
|  | TRINITY_DN1665_c0_g1 | WRKY | PWA70947.1(DNA-binding WRKY [Artemisia annua]) | 0.00 | 1.84 |
|  | TRINITY_DN1725_c0_g1 | WRKY70 | XP_023737392.1(probable WRKY transcription factor 70 [Lactuca sativa]) | 0.00 | 1.82 |
|  | TRINITY_DN7084_c1_g3 | WRKY53 | AJF11719.1(WRKY53 [Chrysanthemum x morifolium]) | 0.00 | 2.90 |
|  | TRINITY_DN27610_c0_g1 | WRKY9 | XP_021988131.1(probable WRKY transcription factor 9 [Helianthus annuus]) | 0.00 | 3.54 |
|  | TRINITY_DN2436_c0_g3 | WRKY57 | XP_021998708.1(probable WRKY transcription factor 57 [Helianthus annuus]) | 0.00 | 1.64 |
|  | TRINITY_DN2436_c0_g1 | WRKY57 | XP_021998708.1(probable WRKY transcription factor 57 [Helianthus annuus]) | 0.00 | 1.40 |
|  | TRINITY_DN30495_c0_g1 | WRKY | PWA68294.1(WRKY domain-containing protein [Artemisia annua]) | 0.00 | 1.38 |
| bHLH | TRINITY_DN6543_c0_g1 | bHLH168 | XP_021995755.1(transcription factor bHLH168 [Helianthus annuus]) | 0.00 | 1.08 |
|  | TRINITY_DN29641_c0_g1 | bHLH51-like | XP_022004867.1(transcription factor bHLH51-like [Helianthus annuus]) | 0.00 | -1.31 |
|  | TRINITY_DN21511_c0_g1 | bHLH61-like | XP_023729735.1(transcription factor bHLH61-like [Lactuca sativa]) | 0.00 | 5.37 |
|  | TRINITY_DN20559_c0_g1 | bHLH30-like | XP_023735519.1(transcription factor bHLH30-like [Lactuca sativa]) | 0.00 | 3.51 |
|  | TRINITY_DN6801_c0_g1 | bHLH106-like | XP_024975551.1(transcription factor bHLH106-like [Cynara cardunculus var. scolymus]) | 0.00 | 2.78 |
|  | TRINITY_DN3789_c0_g1 | bHLH162-like isoform X1 | XP_024973077.1(transcription factor bHLH162-like isoform X1 [Cynara cardunculus var. scolymus]) | 0.00 | -1.25 |
|  | TRINITY_DN24325_c0_g1 | bHLH18-like isoform X1 | XP_023757922.1(transcription factor bHLH18-like isoform X1 [Lactuca sativa]) | 0.00 | 3.66 |
|  | TRINITY_DN35181_c0_g1 | bHLH79 isoform X2 | XP_021983964.1(transcription factor bHLH79 isoform X2 [Helianthus annuus]) | 0.00 | 3.29 |
|  | TRINITY_DN48159_c0_g1 | bHLH93-like | XP_022010123.1(transcription factor bHLH93-like [Helianthus annuus]) | 0.00 | 5.63 |
|  | TRINITY_DN21910_c0_g1 | bHLH30-like | XP_024994780.1(transcription factor bHLH30-like [Cynara cardunculus var. scolymus]) | 0.00 | 1.98 |
|  | TRINITY_DN771_c0_g1 | bHLH74 | XP_024977096.1(transcription factor bHLH74 [Cynara cardunculus var. scolymus]) | 0.00 | 1.46 |
|  | TRINITY_DN25364_c0_g1 | bHLH148-like | XP_023765699.1(transcription factor bHLH148-like [Lactuca sativa]) | 0.00 | 2.01 |
|  | TRINITY_DN2646_c0_g2 | bHLH137 isoform X1 | XP_024970817.1(transcription factor bHLH137 isoform X1 [Cynara cardunculus var. scolymus]) | 0.00 | 1.97 |
|  | TRINITY_DN11476_c0_g1 | bHLH63-like isoform X1 | XP_023747479.1(transcription factor bHLH63-like isoform X1 [Lactuca sativa]) | 0.00 | -1.07 |
| MYB | TRINITY_DN27950_c0_g2 | MYB16-like | XP_024973729.1(transcription factor MYB16-like [Cynara cardunculus var. scolymus]) | 0.00 | 2.95 |
|  | TRINITY_DN15823_c0_g1 | MYB3R-4 isoform X3 | XP_024987199.1(transcription factor MYB3R-4 isoform X3 [Cynara cardunculus var. scolymus]) | 0.00 | 1.57 |
|  | TRINITY_DN13177_c0_g1 | MYB77-like | XP_023728527.1(transcription factor MYB77-like [Lactuca sativa]) | 0.00 | 2.02 |
|  | TRINITY_DN16590_c0_g1 | MYB44-like | XP_021983625.1(transcription factor MYB44-like [Helianthus annuus]) | 0.00 | 1.98 |
|  | TRINITY_DN11745_c0_g1 | MYB124-like isoform X1 | XP_024995020.1(transcription factor MYB124-like isoform X1 [Cynara cardunculus var. scolymus]) | 0.00 | 1.50 |
|  | TRINITY_DN365_c0_g1 | MYB30-like | XP_024978056.1(transcription factor MYB30-like [Cynara cardunculus var. scolymus]) | 0.00 | 1.26 |
|  | TRINITY_DN41979_c0_g1 | MYB4-like | XP_024987600.1(transcription factor MYB4-like [Cynara cardunculus var. scolymus]) | 0.00 | 1.90 |
| bZIP | TRINITY_DN47951_c0_g1 | bZIP60 | XP_023743807.1(bZIP transcription factor 60 [Lactuca sativa]) | 0.00 | -2.02 |
|  | TRINITY_DN29362_c0_g1 | bZIP27-like | XP_024978444.1(bZIP transcription factor 27-like [Cynara cardunculus var. scolymus]) | 0.00 | 3.57 |
|  | TRINITY_DN7880_c1_g1 | bZIP11-like | XP_023769704.1(bZIP transcription factor 11-like [Lactuca sativa]) | 0.00 | 1.84 |
| peroxidase | TRINITY_DN6953_c0_g1 | heme peroxidase | PWA39629.1(heme peroxidase [Artemisia annua]) | 0.00 | 3.74 |
|  | TRINITY_DN5769_c1_g1 | peroxidase superfamily | PWA45869.1(peroxidase superfamily protein [Artemisia annua]) | 0.00 | 1.38 |
|  | TRINITY_DN4908_c0_g1 | peroxidase 4 | XP_023731051.1(peroxidase 4-like [Lactuca sativa]) | 0.00 | 2.59 |
|  | TRINITY_DN285_c0_g1 | peroxidase 64 | XP_023755435.1(peroxidase 64-like [Lactuca sativa]) | 0.00 | 1.79 |
|  | TRINITY_DN13000_c0_g1 | basic peroxidase | XP_022018579.1(basic peroxidase-like [Helianthus annuus]) | 0.00 | 1.06 |
|  | TRINITY_DN13000_c0_g3 | heme peroxidase | PWA72837.1(heme peroxidase [Artemisia annua]) | 0.00 | 4.68 |
|  | TRINITY_DN26976_c0_g1 | peroxidase 17 | XP_021990203.1(peroxidase 17 [Helianthus annuus]) | 0.00 | -1.48 |
|  | TRINITY_DN44479_c0_g1 | peroxidase 20 | PWA74551.1(peroxidase 20 [Artemisia annua]) | 0.00 | -1.60 |
|  | TRINITY_DN21466_c0_g1 | peroxidase 11 | XP_023762048.1(peroxidase 11-like [Lactuca sativa]) | 0.00 | -1.06 |
|  | TRINITY_DN9988_c0_g1 | heme peroxidase | PWA58751.1(heme peroxidase [Artemisia annua]) | 0.00 | 1.40 |
|  | TRINITY_DN21377_c0_g1 | peroxidase 45 | XP_024979499.1(peroxidase 45-like [Cynara cardunculus var. scolymus]) | 0.00 | 1.71 |
|  | TRINITY_DN42109_c0_g1 | peroxidase 72 | XP_023749766.1(peroxidase 72-like [Lactuca sativa]) | 0.00 | 5.24 |
|  | TRINITY_DN9825_c0_g1 | peroxidase 16 | XP_021989613.1(peroxidase 16-like [Helianthus annuus]) | 0.00 | 3.59 |
|  | TRINITY_DN23428_c0_g1 | peroxidase 63 | XP_022005403.1(peroxidase 63-like [Helianthus annuus]) | 0.00 | 2.12 |
| ascorbate peroxidase 3 | TRINITY_DN6366_c0_g1 | ascorbate peroxidase 3 | PWA83814.1(ascorbate peroxidase 3 [Artemisia annua]) | 0.00 | 1.21 |
| HSP | TRINITY_DN20397_c0_g1 | HSP70 | OTF96462.1(putative heat shock protein 70 family, peptide-binding domain protein [Helianthus annuus]) | 0.00 | 3.80 |
| superoxide dismutase | TRINITY_DN77389_c0_g1 | superoxide dismutase | VVA16421.1(PREDICTED: superoxide dismutase [Prunus dulcis]) | 0.00 | -9.20 |
