# Supplementary material for: Integrative physiological and transcriptome analyses provide insights into the Cadmium (Cd) tolerance of a Cd accumulator: Erigeron canadensis
Source: BMC Genomics. 2022 Nov 28;23:778. doi: 10.1186/s12864-022-09022-5 (PMC9703714; doi:10.1186/s12864-022-09022-5)
Supplement: Supplementary file 7 — Additional file 7: Table S6. Primers used for gene expression measurement by qPCR. [file 12864_2022_9022_MOESM7_ESM.doc]

**Table S6** Primers used in experiment of gene expression by qPCR

| **Gene id** | **Forward primer** | **Reverse primer** |
| --- | --- | --- |
| *actin* | TTCCACCCGTGTCCTTGGTA | AGGTGTGGCCCAAGTGACTA |
| TRINITY_DN10006_c0_g1 | TTGGTGGCTCAATGACAGGG | TCAGTGGGGTTTGCACCATT |
| TRINITY_DN35595_c0_g2 | AGCTTTGAGTTCGAGCGTGA | CGGAGCAAGCAATTCCATCG |
| TRINITY_DN5588_c0_g3 | TGTTGGAGGATCGTATGGCG | TGTTGGAGGATCGTATGGCG |
| TRINITY_DN76858_c0_g1 | CTCAGGTCGCATCCCATCAA | AGGGCTATTTTGCCCATGCT |
| TRINITY_DN44511_c2_g1 | CAAAAGGTCCAAGCGCTGTC | ATCCGGGCCTCTTCCTCTAA |
| TRINITY_DN461_c0_g3 | GTTGGGGGTGGTGGTAAGAC | GCACGTCCAGTCTTTGGTGA |
| TRINITY_DN771_c1_g2 | ACAAGACTACACAAATCACCAA | GGCTTCACATCCATTGCATTCT |
| TRINITY_DN9131_c2_g1 | TCAAACAGGCCAAAAGTCGC | GTCAGATTGGGTTGGTCGGA |
| TRINITY_DN5281_c0_g1 | CCGACACCATACGAGCAGAA | CCGCCGGTCAAACTTACTCT |
| TRINITY_DN11992_c0_g1 | TCACAGTGAGTGTAGCACAAAGT | TCCGAGCTGAGTTGAAGAACC |
