# Supplementary material for: Integrative physiological and transcriptome analyses provide insights into the Cadmium (Cd) tolerance of a Cd accumulator: Erigeron canadensis
Source: BMC Genomics. 2022 Nov 28;23:778. doi: 10.1186/s12864-022-09022-5 (PMC9703714; doi:10.1186/s12864-022-09022-5)
Supplement: Supplementary file 8 — Additional file 8: Figure S2. qRT‒PCR verification of the expression levels of 10 DEGs (five upregulated and four downregulated) determined by RNA-seq. The left y-axis represents the relative expression level, and the right y-axis represents the RNA-seq data (FPKM value). The box with error bars indicates the qRT‒PCR result, and the oblique line represents the FPKM value of gene expression. [file 12864_2022_9022_MOESM8_ESM.doc]

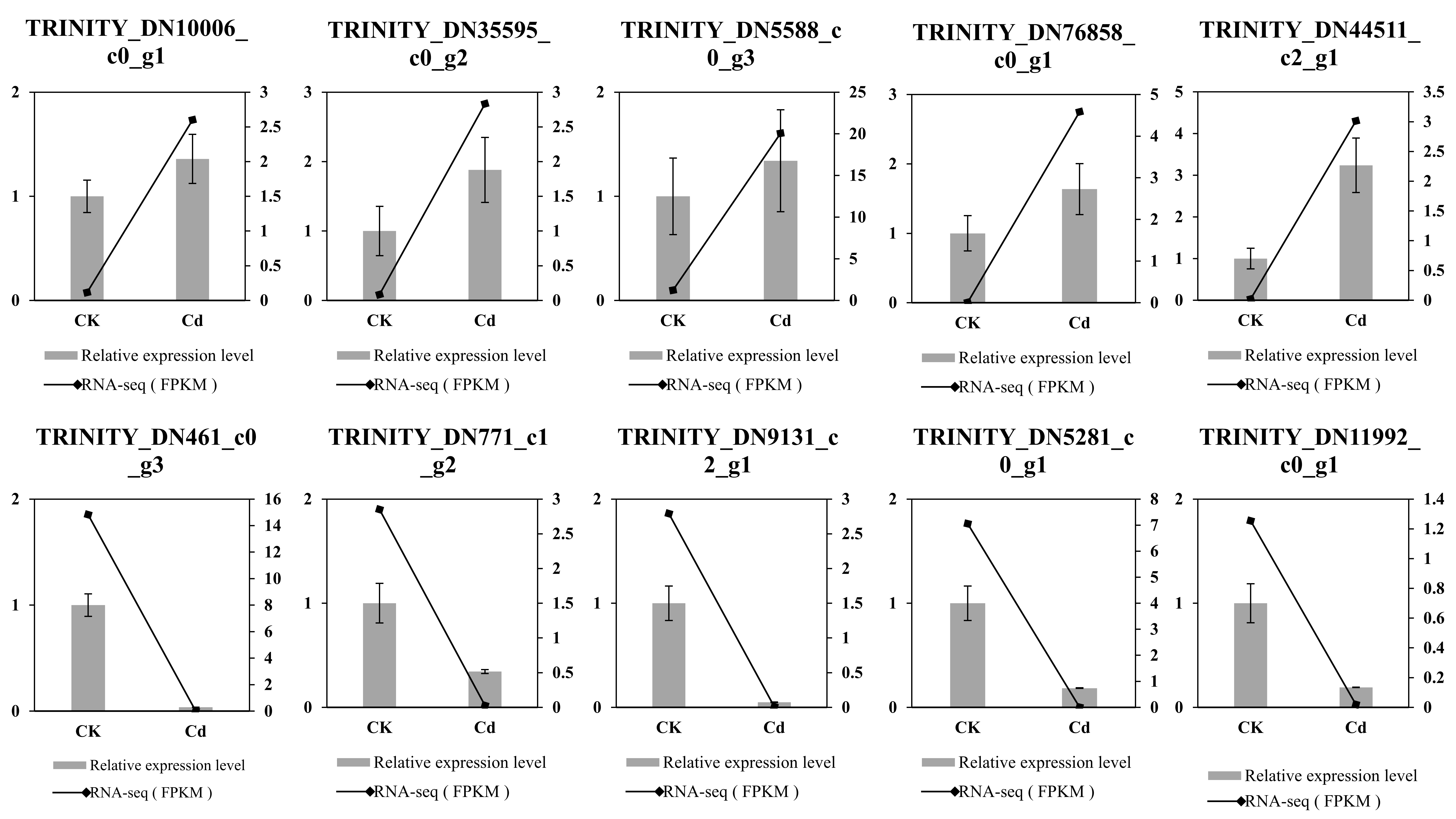


Figuer S2. qRT‒PCR verification of the expression levels of 10 DEGs (five upregulated and four downregulated) determined by RNA-seq. The left y-axis represents the relative expression level, and the right y-axis represents RNA-seq (FPKM). The box with error bars indicates the qRT‒PCR result, and the oblique line represents the FPKM value of gene expression.
